# *Spiroplasma eriocheiris* Adhesin-Like Protein (ALP) Interacts with Epidermal Growth Factor (EGF) Domain Proteins to Facilitate Infection

**DOI:** 10.3389/fcimb.2017.00013

**Published:** 2017-01-26

**Authors:** Libo Hou, Yuhan Liu, Qi Gao, Xuechuan Xu, Mingxiao Ning, Jingxiu Bi, Hui Liu, Min Liu, Wei Gu, Wen Wang, Qingguo Meng

**Affiliations:** ^1^Jiangsu Key Laboratory for Biodiversity and Biotechnology and Jiangsu Key Laboratory for Aquatic Crustacean Diseases, College of Life Sciences, Nanjing Normal UniversityNanjing, China; ^2^Co-Innovation Center for Marine Bio-Industry Technology of Jiangsu ProvinceLianYungang, China

**Keywords:** *Spiroplasma eriocheiris*, adhesin-like protein, 3T6-Swiss albino, epidermal growth factor, fibulin7

## Abstract

*Spiroplasma eriocheiris* is a novel pathogen found in recent years, causing the tremor disease (TD) of Chinese mitten crab *Eriocheir sinensis*. Like *Spiroplasma mirum, S. eriocheiris* infects the newborn mouse (adult mice are not infected) and can cause cataract. Adhesion-related protein is an important protein involved in the interaction between pathogen and host. In this study, the Adhesin-like Protein (ALP) of *S. eriocheiris* was detected on its outer membrane by using immune electron microscopy, and was found to be involved in the bacterium's infection of mouse embryo fibroblasts (3T6-Swiss albino). Yeast two-hybrid analysis demonstrated that ALP interacts with a diverse group of mouse proteins. The interactions between recombinant partial fibulin7 (FBLN7; including two epidermal growth factor [EGF] domains) and ALP were confirmed by Far-western blotting and colocalization. We synthetized the domains of FBLN7 [EGF domain: amino acids 136–172 and complement control protein (CCP) domain: 81–134 amino acids], and demonstrated that only EGF domain of FBLN7 can interact with ALP. Because the EGF domain has high degree of similarity to EGF, it can activate the downstream EGFR signaling pathway, in key site amino acids. The EGFR pathway in 3T6 cells was restrained after rALP stimulation resulting from competitive binding of ALP to EGF. The unborn mouse, newborn mouse, and the adult mouse with cataract have a small amount of expressed FBLN7; however, none was detected in the brain and very little expression was seen in the eye of normal adult mice. In short, ALP as a *S. eriocheiris* surface protein, is critical for infection and further supports the role of ALP in *S. eriocheiris* infection by competitive effection of the EGF/EGFR axis of the target cells.

## Importance

*S. eriocheiris* has been previously identified as a novel pathogen of *E. sinensis* and caused mass mortality in aquaculture. It is interesting that *S. eriocheiris* has the ability to infect 3T6 cells other than invertebrates such as crustaceans. However, how it infects the host cells and its cellular molecular pathogenesis is poorly understood. Adhesin-like protein (ALP) plays an important role in this process. We demonstrated that *S. eriocheiris* ALP is critical for the bacterial infection of host cells, and may be important in finding ways to prevent host cell infection.

## Introduction

*Spiroplasma eriocheiris* is a causative agent of the tremor disease (TD) of Chinese mitten crab *Eriocheir sinensis* (Wang et al., 2003, 2011), and a novel pathogen of aquatic crustaceans including *Procambarus clarki, Penaeus vannamei, Macrobrachium rosenbergii*, and *Macrobrachium nipponense* (Bi et al., 2008; Liang et al., 2011; Xiu et al., 2015). Historically, the *Spiroplasma* spp. were known to associate with only insects, ticks, and plants; however, recent isolations from crustaceans are beginning to change our understanding of the host range (Regassa and Gasparich, 2006). Phylogenetic analysis of the 16S rDNA gene sequences of *S. eriocheiris* showed that this *Spiroplasma* strain had a close relationship with *S. mirum* (Wang et al., 2004a). *S. mirum* isolated from the rabbit tick (*Haemaphysalis leporispalustris*) can infect vertebrates (Kirchhoff et al., 1981; Tully et al., 1982). It induced the presence of suckling mouse cataract agent (SMCA) (Zeigel and Clark, 1974). Similarly, *S. eriocheiris* was detected in the brain of embryonated chickens (Wang et al., 2003), and it was shown to infect the newborn mouse and cause cataract; adult mice are not infected (Figure S1). Therefore, it is important to study how *S. eriocheiris* infects mouse cells and its cellular molecular pathogenesis.

Presently, several lines of evidence suggest that host-pathogen interactions could be a prerequisite for invasion and colonization of bacteria (Wayadande and Fletcher, 1998; Ammar and Hogenhout, 2005; Suzuki et al., 2006; Ojogun et al., 2012; Kahlon et al., 2013). *Mollicutes* lack a cell wall (Stülke et al., 2009), and it is well-established that successful colonization of the host cells requires adhesion as the first step. Adhesion of *Mycoplasma* and *Spiroplasma* to host cells is a prerequisite for colonization by the parasite and subsequent infection; hence, adhesion-related proteins play an important role in this process (Rottem, 2002; Balish et al., 2003). The loss of adhesion capacity by mutation results in loss of infectivity, while reversion to the cytadhering phenotype is accompanied by regaining infectivity and virulence (Krause et al., 1982, 1983). Entry of *S. eriocheiris* involves interaction between the pathogen and host that induces cellular signaling events. However, few studies have focused on how the adhesion-related protein interacts with the host protein. We have been studying the role of *S. eriocheiris* adhesin-like protein (ALP) in the infection of *E. sinensis* (Meng et al., 2010). However, the role and the host interaction proteins of ALP in the process of *S. eriocheiris* infect mouse cells have not yet been defined.

In this study, we demonstrated that ALP is located in the *S. eriocheiris* membrane and we used a yeast (*Saccharomyces cerevisiae*) two-hybrid (Y2H) assay to identify molecular ALP-host interaction proteins with distinct molecular functions, suggesting that it plays an important and complex role in the process of bacterial entry into the host cell.

## Materials and methods

### *S. eriocheiris* culture, host cell culture, and treatment

*S. eriocheiris* was isolated from *E. sinensis* with TD using the methods described by Wang et al. (2004b) and cultured in R2 medium at 30°C (Moulder et al., 2002). 3T6-Swiss albino (3T6) cells were purchased from the Type Culture Collection cell bank of the Chinese Academy of Sciences Committee (Shanghai, China) and Human cervical epithelial adenocarcinoma (HeLa) cells from Professor Chen's lab. The cells were cultured in Dulbecco's modified Eagle's medium (DMEM), (Wisent, Canada) complete medium supplemented with 10% fetal bovine serum (FBS), 0.15% NaHCO_3_, 0.45% glucose, 4 mM L-Glutamine (Wisent, Canada), and antibiotics (100 U ml^−1^ penicillin, 100 U ml^−1^ streptomycin) at a suitable pH of 7.20–7.40.

When the cell cultures grew to >70% confluence, they were treated in the following ways: (1) 3T6 cells were treated with *S. eriocheiris* (*S. eriocheiris* groups) for 36 h; (2) 3T6 cells were infected with *S. eriocheiris*, which was treated with anti-ALP (1:2000) for 1 h at 30°C (*S. eriocheiris*+Anti-ALP groups), and incubated for 36 h; (3) 3T6 cells were infected with *S. eriocheiris*, which was treated with pre-immume serum for 1 h at 30°C (*S. eriocheiris*+serum groups), and incubated for 36 h; (4) 3T6 cells were treated with recombinant ALP (5 μg/ml) for 0, 15, 30, 45, and 60 min (using 0 min as control); (5) 3T6 cells were treated with EGF (20 ng/ml) and using different concentrations of recombinant ALP (0, 0.5, 1, 5, and 10 μg/ml) for 15 min, cells without any treatment served as a control; (6) HeLa cells were co-transfected with plasmids encoding EGFP-ALP and DsRed-Fiblin7, using X-tremeGENE HP DNA Transfection Reagent (Roche) according to the supplier's instructions. The all host cells were culture with same conditions 37°C and 5% CO_2_.

### Antibodies

Rabbit anti-ALP antibodies and polyclonal antibody against *S. eriocheiris* have been described previously (Meng et al., 2010). Other antibodies used in this study were mouse anti-Glyceraldehyde-3-phosphate dehydrogenase (GAPDH), mouse-anti extracellular regulated protein kinases (ERK), Phospho-mouse-anti extracellular regulated protein kinases (p-ERK), mouse-anti Ras homolog gene family, member A (RhoA), mouse-anti Phospho-Ras homolog gene family, member A (p-RhoA), mouse-anti β-Catenin, mouse-anti Phospho- mouse β-Catenin, mouse anti Akt, mouse-anti Phospho-Akt, anti-GST tag, and Alexa Fluor 488-labeled Goat Anti-Rabbit IgG (Beyotime, China) and Goat anti-musculus fibulin7 (FBLN7) (Cell Signaling, USA). All antibodies used for immunofluorescence were tested by the vendor to ensure the specificity and confirmed by western blotting, immunofluorescent microscopy, or both.

### Immuno-EM

For Immuno Gold Electron Microscope (EM), the *S. eriocheiris* cells bound to EM grids were treated with Triton solution containing 0.3% Triton X-100, fixed using 3% paraformaldehyde, and 0.1% glutaraldehyde in phosphate buffer saline (PBS) for 10 min at RT, and washed three times by PBS. The cells structures on grids were treated by applying 10-fold diluted ALP polyclonal antibody in PBS containing 2% BSA, and subsequently washed five times using PBS. Then, the cell preparations were treated by applying 10-fold diluted gold-labeled secondary antibody (5 nm colloidal-gold-labeled goat antibody; Sigma) in PBS containing 2% BSA for 30 min at RT, washed five times, and then stained by 2% molybdate. The samples were examined using an H-7650 transmission electron microscope (Hitachi, Japan).

### Membrane protein and cytoplasm protein of *S. eriocheiris* preparation

Membranes and cytoplasm were isolated from the washed cells as described by Michael Salman (Salman and Rottem, 1995). Briefly, washed bacteria were suspended in a 10 mM NaCl solution containing 0.5 mM β-mercaptoethanol and treated for 3 min at 4°C in a W-350 Heat Systems sonicator operated at 50% duty cycles and an intensity of 200 W. Intact cells were removed by centrifugation at 5000 × g for 5 min, and membranes were then collected by centrifugation at 34,000 × g for 40 min. The supernatant containing cytoplasmic protein and the centrifuged pellet were each washed once and resuspended in 10 mM NaC1 solution. Protein concentration was determined by the BCA method with bovine serum albumin as a standard. The proteins were detected by 12% SDS-polyacrylamide gel electrophoresis (SDS-PAGE).

### Immunofluorescence experiment

In the cell challenge test, samples were collected every 2 h between 30 and 48 h post challenge and washed three times with PBS. Then, the 3T6 cells were fixed with 4% paraformaldehyde and permeated with PBS containing 0.1% Triton X-100 for 30 min. After the cells were incubated with 3% BSA in PBST for 30 min, the ALP polyclonal antibody, incubated with *S. eriocheiris*, was used to inhibit adhesion to and infection of 3T6 cells. The cells infected with *S. eriocheiris* (cells without treatment and *S. eriocheiris* treated with pre-immume serum served as the control. Then, the cells were incubated with Alexa Fluor 488-conjugated goat anti-rabbit IgG secondary antibody (Invitrogen, USA) and examined using a Ti-s inverted phase-contrast microscope (NikonH600L, Japan).

### Yeast two-hybrid system

A Clontech Matchmaker Two-Hybrid system, yeast media and supplements, vectors, and yeast transformation system was used.

### Cloning of *S. eriocheiris* ALP gene and autoactivation test

The full-length sequence of *ALP* (GenBank accession number GU046560) gene was amplified by PCR from *S. eriocheiris* genomic DNA using the specific primers (ALP; Table 1). After site-directed mutagenesis (ALP-M), it was cloned into the Sal I/EcoR I site of pGBKT7 vector containing the GAL4 DNA-BD.

**Table 1:** **Primers used for cloning the EIF2, FBLN7, and ALP**.

**Name**	**Sequence (5′–3′)**
EIF2-F	GAAGGATCCATGGCATCGGCGGTGGTTG
EIF2-R	GTGGCGGCCGCTTTCTCACACGTCACTAGCC
FBLN7-F	GAATTCTTCCACCTGAGCAGCACCACG
FBLN7-R	GAGCTCACGGCTGTTCCCACCCACGAT
ALP41-F	CCGAATTCATGTTGGCCTGTTCAACT
ALP41-R	ATGTCGACTTAGTTATTTTCATAATACCAAATTCC
ALP41-M-F	TAATGCGTTAGTTAGTGACC
ALP41-M-R	ATTTCCCACCACTCATCACT

To examine whether the bait (ALP) autonomously activated (autoactivated) the reporter genes in yeast (*S. cerevisiae*) strain AH109 in the absence of a prey protein, AH109 cells were transformed with bait plasmid pGBKT7-ALP and plated on SD/-Trp medium, transformed with pGBKT7-empty vector as the negative and pCL1 vector as the positive controls. The results were obtained using β-galactosidase by Colony-lift Filter Assay (Lin et al., 2005; Liu et al., 2010). Briefly, AH109 yeast cells transformed with empty plasmid, and pGBKT7-ALP positive control plasmid were cultured on SD/-Trp agar plates at 30°C for 2–4 days. A clean and dry filter was placed over the surface of the agar plate until evenly wetted, carefully lifted off, and transferred to a pool of liquid nitrogen for 10 s. After thawing at room temperature, the filter was placed with colonies facing up on another filter presoaked in Z buffer/X-gal solution and incubated at 30°C for up to 8 h, checking periodically for the appearance of blue colonies.

### Yeast two-hybrid assay

The Mate & Plate™ Library-Universal Mouse (Normalized) Clonetech, a high-complexity cDNA library cloned into the yeast GAL4 activation domain (GAL4-AD) vector pGADT7-Rec and pre-transformed into *S. cerevisiae* host strain Y187, was mated with bait strain AH109 containing pGBKT7-ALP according to the manufacturer's protocol. Positive clones expressing prey proteins interacting with ALP (bait) were selected on minimal synthetically defined (SD) quadruple-dropout (QDO) medium (SD medium without Ade, His, Leu, and Trp [SD/-Ade/-His/-Leu/-Trp]) supplemented with 5-bromo-4-chloro-3-indolyl-α-D-galactopyranoside (X-α-Gal) and aureobasidin A (QDO/X/A). The colony-lift filter assay was also used. Normal-sized blue colonies were segregated three times on SD double-dropout (DDO; SD medium without Leu and Trp [SD/-Leu/-Trp]) plates containing X-Gal (DDO/X), and the prey cDNA inserts were amplified by colony PCR.

### Confirmation of positive interactions by cotransformation

The prey plasmid responsible for positive interactions was rescued from segregated colonies using an Easy yeast plasmid isolation kit (Clontech, USA), transformed into *Escherichia coli* DH5α, and isolated. To distinguish positive from false-positive interactions, AH109 yeast cells were cotransformed with bait (pGBKT7-ALP) and prey plasmids. The positive interactions were confirmed by selection on SD/-Trp/-Leu and detected through β-galactosidase by Colony-lift Filter Assay.

### Recombinant proteins expression and synthesis of peptides fragment

To confirm interactions between bait and prey proteins identified by Y2H, the *Mus musculus* E74-like factor2 (EIF2, full length) and fibulin7 (FBLN7, 211-382aa, containing two EGF domains) were cloned using the specific forward primer and the reverse primer listed in Table 1. The PCR products were each digested with restriction enzymes and cloned into pGEX4T-1 vectors (containing a GST tag), namely, pGEX-EIF2 and pGEX-FBLN7. Afterward, *E. coli* BL21 (DE3) was transformed with the resulting recombinant plasmid for IPTG (isopropyl β-D-1-thiogalactopyranoside)-induced recombinant expression (final IPTG concentration of 0.5 mM). The induction temperature for protein production was 37°C. The proteins were detected on 12% SDS-PAGE and western blots.

FBLN7 is 440 amino acids long, contains four distinct domains: a complement control protein (CCP) domain (amino acids 81–134), and three EGF domains (amino acids 136–172, 225–270, 174–320; de Vega et al., 2007). The recombinant FBLN7 protein (amino acids 211–382) contains two distinct EGF domains. The other two domains, CCP domains (amino acids 81–134) and EGF domains (amino acids 136–172), were artificially synthesized (China Peptides Shanghai China).

### Far-western blotting analysis

To confirm ALP interaction with the proteins EIF2 and FBLN7, far-western blotting analysis was performed. Because the recombinant protein contains a GST tag, GST tag protein was used as a negative control. Briefly, EIF2, FBLN7, and GST tag were separated on 12% SDS-PAGE and transferred onto a PVDF membrane. The membrane was then incubated with wash buffer (2 M Tris, pH 7.5 containing 500 mM NaCl, and 0.1% (v/v) Tween-20) containing 5% BSA for 2 h at room temperature to block non-specific protein binding. The membrane was washed with wash buffer, and incubated with 25-μg His-ALP in 5 mL of wash buffer containing 5% BSA overnight at 4°C. After washing, the integrin-bound protein band was incubated with primary antibodies (rabbit anti-ALP serum), and then were diluted 1:2500 in wash buffer containing 5% BSA for incubation overnight at 4°C. Following washing, the blots were incubated with HRP-conjugated anti-rabbit IgG (diluted 1:5000 in wash buffer) for 1 h at room temperature (Egan et al., 2006). Immunoreactive bands were visualized by the enhanced chemiluminescence (ECL) system (Vazyme China). Far-western blotting analysis was also used to confirm ALP interaction with synthetic EGF and CCP. The EGF and CCP protein were separated on 15% SDS-PAGE and transferred onto a PVDF membrane; the other methods were followed as described above.

### Colocalization

Colocalization was used to further confirm interactions between bait and prey proteins identified by Y2H in mammalian cells. Briefly, two mammalian expression vectors, pEGFP-N2, and pDsRed-Monomer-N1, which encode an enhanced green fluorescent protein (EGFP) tag and a DsRed (a red fluorescent protein) tag, respectively, were used for generation of C-terminal EGFP-ALP and DsRed-FBLN7 fusion proteins (Tseng et al., 2010; Lin et al., 2015). The ALP was cloned in-frame upstream of the EGFP tag of pEGFP-N2, while the FBLN7 gene was amplified from GADT7-FBLN7 (containing two distinct EGF domains) and cloned in-frame upstream of the DsRed tag of pDsRed-Monomer-N1. HeLa cells at high confluence were cotransfected with plasmids encoding EGFP-ALP and DsRed-FBLN7, using the X-treme GENE HP DNA Transfection Reagent (Roche) according to the supplier's instructions. To study the colocalization at 1 day post-transfection, cells were washed with PBS, fixed with 4% paraformaldehyde in PBS for 15 min at room temperature, followed by washing the cells with PBS. The cells were examined and recorded using a confocal microscope (Nikon TI-E-A1R; Nikon) and representative cells were selected and photographed.

### Western blotting analysis

After treatment, cells were briefly washed with cold PBS. While on ice, they were lysed in RIPA buffer (50 mM Tris, pH 7.2; 1% sodium deoxycholate; 150 mM NaCl; 0.1% sodium dodecyl sulfate; 10 mM NaF; 1% Triton-X 100; 1 mM Na_3_VO_4_; protease inhibitor cocktail [1:1000]). Lysates were sonicated for 10 s and centrifuged at 13,000 × g for 10 min at 4°C. Protein concentration was determined by the BCA method with bovine serum albumin as the standard. Equivalent amounts of protein were separated on 12% SDS-PAGE and transferred onto PVDF membranes. Membranes were incubated with PBS containing 0.05% Tween 20 and 5% BSA to block nonspecific binding and were incubated with primary antibodies (ERK antibody, p-ERK antibody, RhoA antibody, p-RhoA antibody, β-Catenin antibody, p-β-Catenin antibody, Akt antibody, p-Akt antibody, and GAPDH antibody), then treated with appropriate secondary antibodies conjugated to horseradish peroxidase. After this, the bands were visualized using ECL. All the Western blotting analysis were repeated on several occasions, and the gray value of the bands was detected with the ImageJ software.

### Protein expression tendency of FBLN7

For the developmental analysis of FBLN7 protein expression, we obtained the brains and eyes of mouse from different stages of mouse development. Animals' brains and eyes were obtained at prenatal 3 days; postnatal 6, 12, 24, 60 h; and postnatal 60 days (adult). The mice afflicted with cataracts were treated as described previously (Riederer and Matus, 1985). Previous studies have confirmed that the expression quantity of FBLN7 in the placenta was the highest. Therefore, we selected this tissue as a positive control. The samples were cut into pieces before protein extraction. The method of protein extraction was listed above. The FBLN7 expression was analyzed by western blotting as above. This study was carried out in accordance with the recommendations of Institutional Animal Care and Use Committee of the Nanjing Normal University [SYXK (Jiangsu) 2015–0028].

### Statistical analysis

Data were analyzed using the SPSS general linear models (GLM) procedure (SPSS 16.0, Chicago, IL, USA) to test for significant differences among treatments. If a significant (*P* < 0.05) difference was found, a Duncan's multiple range test (Duncan, 1955) was used to rank the means. All data are presented as mean ± S.D (standard deviation) of three replicates.

## Results

### Surface localization of ALP

**Figure 1:**
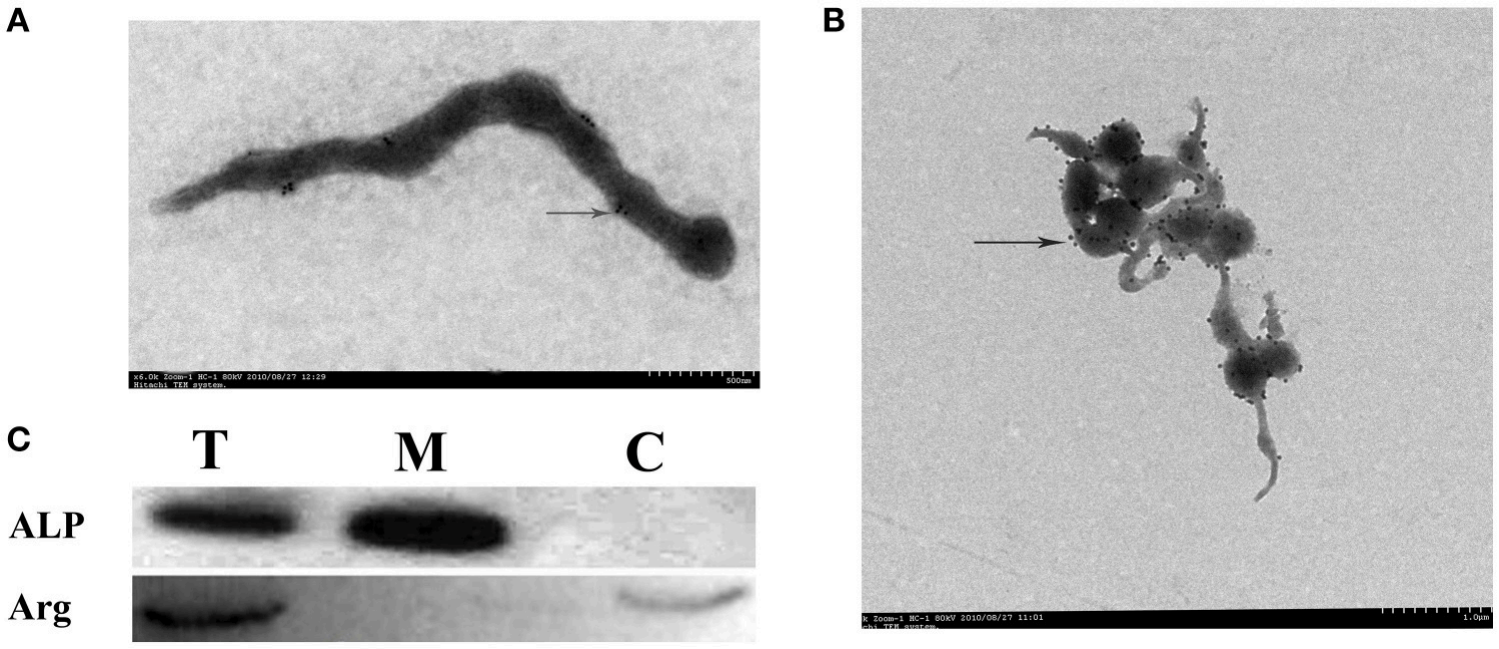
**ALP localizes on the surface of *S. eriocheiris*. (A,B)** Immunogold labeling for ALP on the *S. eriocheiris*. An arrowhead indicates the ALP on the surface of *S. eriocheiris*. **(C)** Western blotting was used to analyze the location of ALP, three lanes were represented; i.e., all proteins (T), membrane proteins (M), and cytoplasmic protein (C) of *S. eriocheiris*, respectively.

Firstly, ALP was verified as a surface protein. Transmission EM (TEM) immuno-gold labeling showed that native ALP localizes on the surface of the *S. eriocheiris* (Figures 1A,B). After preparation of membrane protein, cytoplasmic protein, and total protein of *S. eriocheiris*, ALP antiserum (dilute1:2000) was used to determine whether immunoaccessible domains were present on the *S. eriocheiris* surface using western blotting. In agreement with TEM (Figure 1C), the results showed that the ALP signal was detected in the lanes of total protein from *S. eriocheiris* (T) and membrane proteins (M), but not in cytoplasmic protein (C), thus indicating that ALP is exposed on the surface of *S. eriocheiris*. Arginine deaminase (Arg), as has been previously demonstrated, was one of the *S. eriocheiris* cytoplasmic proteins used as a negative control. The Coomassie stained PAGE gels used in the Western blots in a Supplementary Figure as loading control (Figure S2).

### ALP antibody inhibition of *S. eriocheiris* infection

**Figure 2:**
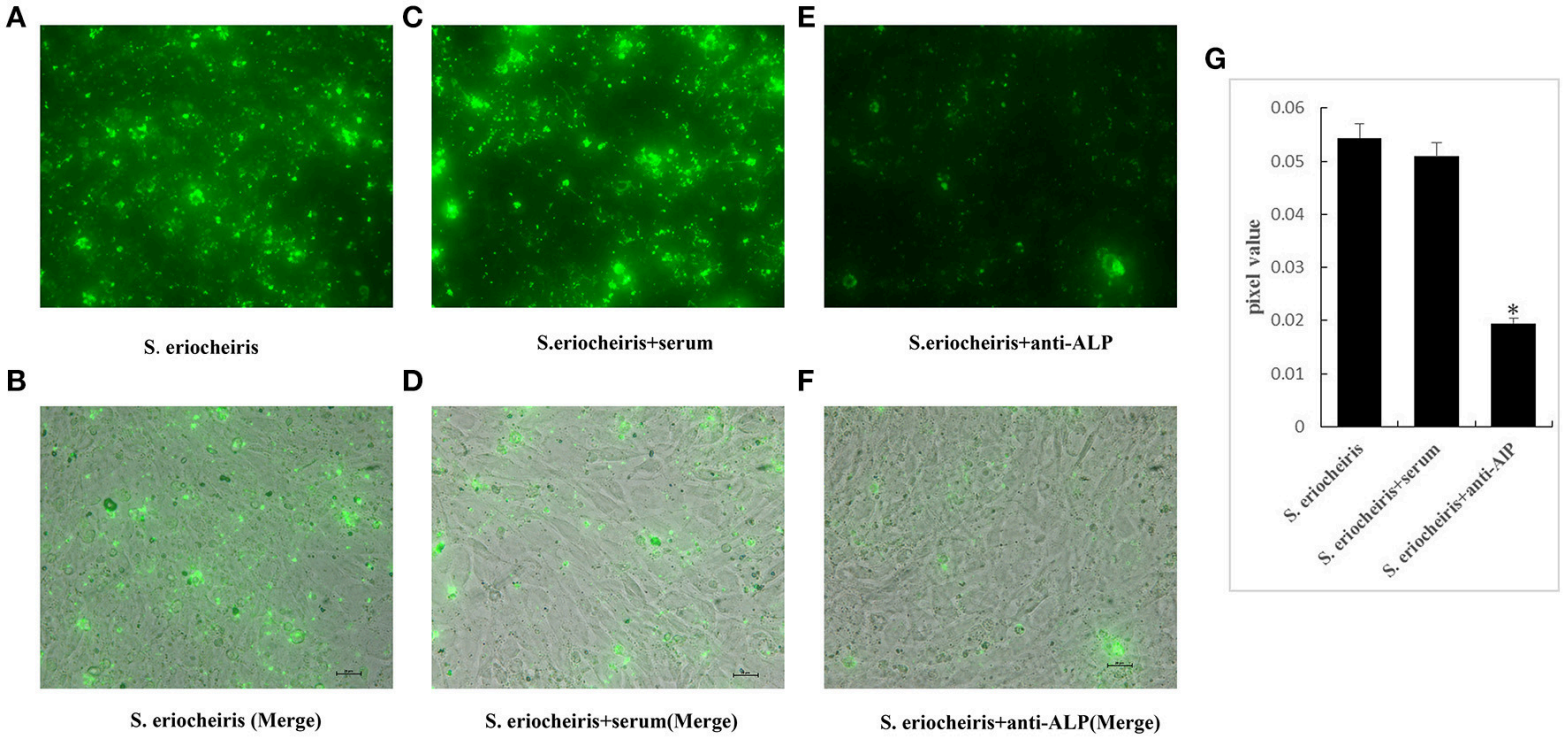
**Pretreatment of *S. eriocheiris* with anti-ALP reduces infection of 3T6 cells**. Cells treated with *S. eriocheiris* (*S. eriocheiris* groups) and *S. eriocheiris* were incubated with anti-ALP or pre-immume serum (*S. eriocheiris*+anti-ALP groups or *S. eriocheiris*+serum) for 36 h. After removal of unbound bacteria, bacteria attached to the host cells were labeled with Alexa Fluor 488-labeled Goat Anti-Rabbit IgG and assessed using confocal microscopy. The green fluorescence represents the bacteria. **(A,B)** represent the *S. eriocheiris* groups: **(A)** green fluorescence showing the bacteria, and **(B)** the host cells merging with the green fluorescence. **(C,D)** represent the *S. eriocheiris*+serum groups: **(C)** green fluorescence indicates the bacteria, and **(D)** the host cells merging with the green, fluorescently-labeled bacteria. **(E,F)** represent the *S. eriocheiris*+anti-ALP groups: **(E)** green fluorescence indicates the bacteria, and **(F)** the host cells merging with the green, Bars, 20 μm. **(G)** The Panels **A,C,E** unit area pixels value were measured. Statistical significance is indicated with an asterisk (^*^).

To directly show that ALP plays an important role in the process of *S. eriocheiris* infection of 3T6 cells, an immunofluorescence experiment was conducted. The *S. eriocheiris* were incubated with anti-ALP (dilute1:2000) for 1 h at 30°C, and then bacterial adhesion and infection of 3T6 cells were monitored. The results showed that the quantities of *S. eriocheiris* infection within the 3T6 cell interior were significantly lower than that in the control group at 36 h post-inoculation (Figure 2).

### Analysis of *S. eriocheiris* ALP interactions with host using a yeast two-hybrid system

To confirm that the bait (ALP) did not autonomously activate (autoactivate) the reporter genes in yeast strain AH109 in the absence of a prey protein, AH109 cells were transformed with bait plasmid pGBKT7-ALP and plated on SD without Trp (SD/-Trp), using the transformed pGBKT7-empty vector as the negative and pCL1 as the positive controls. The results were detected by colony-lift filter β-galactosidase assay. No blue reaction was detected in colonies with bait plasmid pGBKT7-ALP and pGBKT7-empty, confirming a lack of autoactivation by ALP.

After the yeast two-hybrid screening using yeast mating, a total of 137 yeast colonies with blue color and normal size were observed and selected from SD/-Trp/-Leu/-Ade/-His/X/A (QDO/X/A) plates for identification of potential positive clones that exhibited bait and prey protein-protein interactions.

### Confirmation of interactions by cotransformation

**Figure 3:**
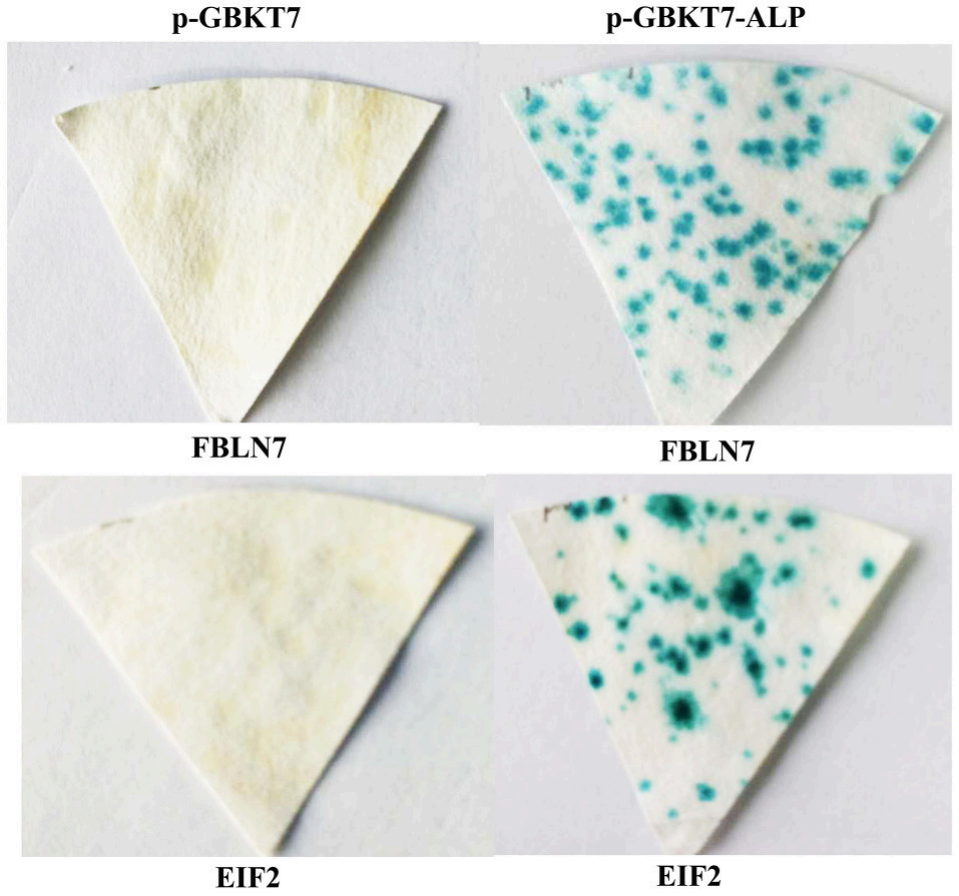
**Confirmation of positive interactions in yeast by cotransformation**. Y2HGold yeast cells were cotransformed with bait plasmid pGBKT7-ALP and prey plasmid pGADT7-FBLN7 and -EIF2. As a control, pGBKT7 (empty vector) was used to cotransform with each prey plasmid. The positive interactions were confirmed.

To confirm the true interactions, 23 colonies were randomly chosen from 137 yeast colonies with blue color. The cotransformation assays were performed in yeast with prey plasmids, and the results showed that all of the selected colonies were a result of interactions with the prey plasmids in yeast. After segregation of the colony three times, yeast colony PCR was performed. This was followed by DNA sequencing to eliminate the duplicate clones and identify interacting targets. Twelve different potentially interacting mouse proteins were identified (Table 2) and the DNA sequencing data for interacting target proteins are provided in Supplementary Data. Interactions in this experiment were confirmed with all these prey proteins (Figure 3, showing interactions of ALP with two prey proteins: FBLN7 and EIF2).

**Table 2:** **Positive hit of the screening**.

**Bait**	**Prey library**	**Identical colonies**	**Positive gene name**	**Positive gene identified**	**NCBI accession NMmeber**	**Gene Code match**
pGBKT7-ALP41	Universal Mouse (Normalized) Clonetech No. 630482	23	ALP1/	EUCOMM	JN956676.1	NO
			ALP 9			
			ALP 2	KLHL 10	NM025727.3	YES
			ALP 3	PNPLA 8	NM026164.2	YES
			ALP 4	NIPAL 3	NM028995.3	NO
			ALP 5	FBLN 7	NM024237.4	YES
			ALP 7	CAR 8	NM007592.3	YES
			ALP 10	GSg 1	NM010352.2	YES
			ALP 12	LMPA 2	NM053261.2	YES
			ALP 13	COPS 5	NM013715.2	YES
			ALP 14	XPNPEP 1	NM133216.3	YES
			ALP 18	MCCC 2	NM030026.2	YES
			ALP 19	SLC34A2	NM011402.3	NO
			ALP 20	EIF 2	NM023502.1	YES
			ALP 23	D3Ertd254e	NM001101478.1	NO
			ALP 29	KCNJ 15	NM001271695.1	NO
			ALP 28	Chromosome 18	AC102081.15	NO
			ALP 27	YrDC	NM153566.2	YES
			ALP 25	BCA clone		NO
			ALP 30	Chromosome 3	AC093365.10	NO
			ALP 31	TEX 38	NM029196.1	NO
			ALP 36	FOXred 1	NM172291.1	YES
			ALP 37	YIF1A	NM026553.4	NO

### Recombinant protein expression

Using yeast two-hybrid screening, several proteins were identified that interacted with *S. eriocheiris* ALP. From among them, two proteins, EIF2 (full length) and FBLN7 (amino acids 211–382), were selected to clone and obtain recombinant protein expression for further studies. After being expressed, recombinant EIF2 and FBLN7 had an apparent molecular weight of around 95 and 43 kDa, respectively (Figure S3). The recombinant proteins were purified using a Gel Extraction kit according to the manufacturer's protocol.

### Confirmation of interaction of identified proteins with ALP

**Figure 4:**
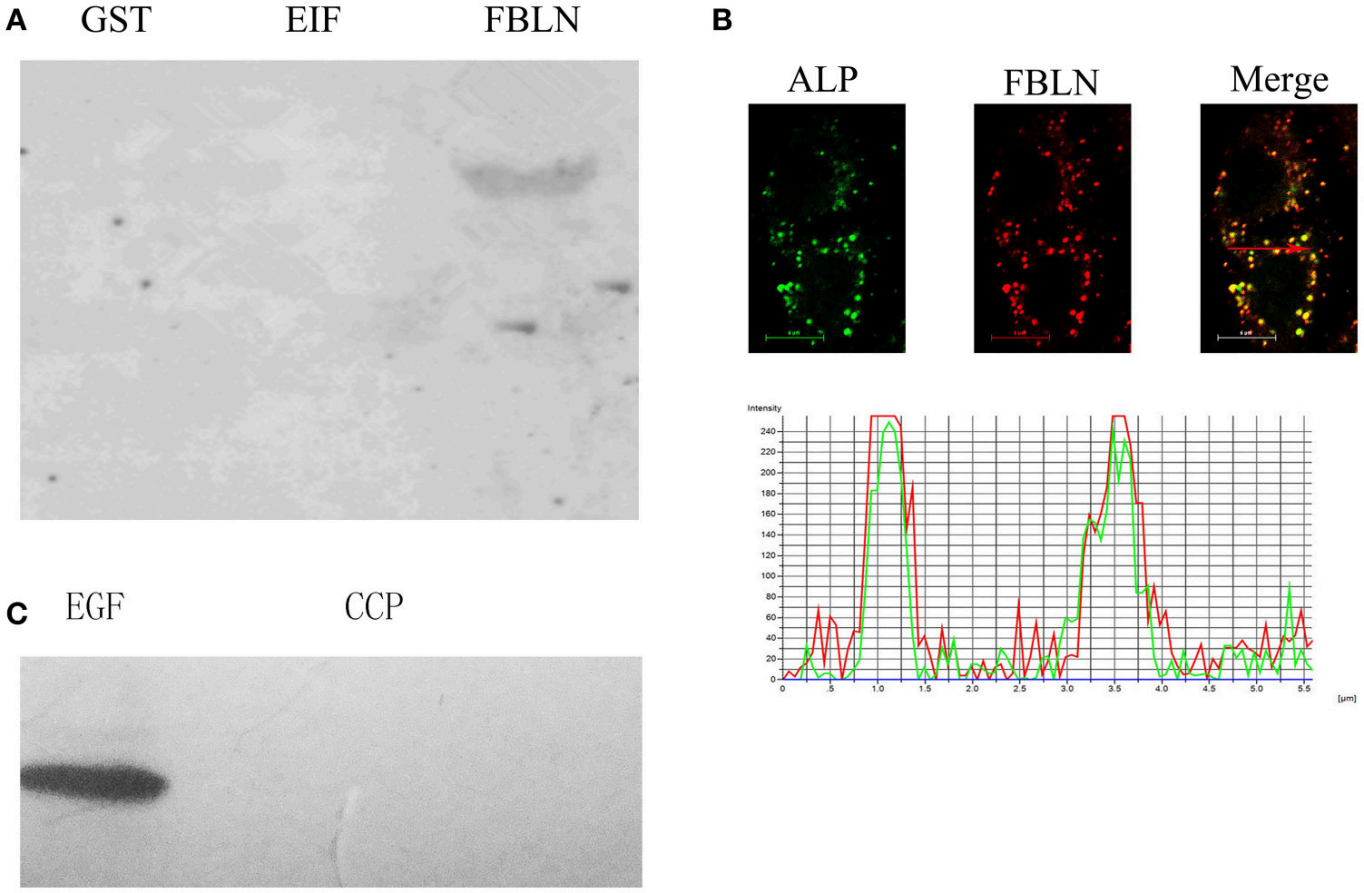
**The interaction between ALP and FBLN7. (A)** The interaction between ALP and the domain of FBLN7 was detected by far-western blotting. The GST lanes are the control, and EIF and FBLN lanes represent the recombinant EIF and FBLN7, respectively. **(B)** Colocalization of FBLN7 with *S. eriocheiris* ALP in HeLa cells. Full-length ALP and partial sequence (amino acids 211–382) FBLN7 cDNAs were cloned into pEGFP-N2 and pDsRed-monomer N1 and transfected into undifferentiated HeLa cells as described in the Materials and Methods Section. Twenty-four hours after transfection, cells were fixed and processed for imaging. Fluorescence microscopy and intensity profiles of HeLa cells, ALP (green), and FBLN7 (red), show colocalization of *S. eriocheiris* ALP with FBLN7. The red arrows in the fluorescence images indicate the areas selected for fluorescence intensity profile analyses, which are displayed in graph form. The X-axis shows the position along the line (pixels), and the Y-axis shows the fluorescence intensity. Bars, 6 μm. **(C)** The interaction between ALP and the EGF domain of FBLN7 was detected by far-western blotting. The EGF and CCP represent the EGF domain and CCP domain of FBLN7, respectively.

In order to further examine the interaction of ALP with EIF2 and FBLN7, Far-western blotting analysis was performed. The results confirmed a direct interaction between ALP and FBLN7 (amino acids 211–382, contain two GEF domains; Figure 4A), none interaction between ALP and GST tage and EIF2. Interactions between ALP and FBLN7 were examined in mammalian (HeLa) cells by cotransfection assay. Immunofluorescence microscopy revealed that ALP and FBLN7 (amino acids 211–382, contain two GEF domains) were colocalized in HeLa cells, and exhibited the strongest colocalization (Figure 4B). The negative control was cotransfected using EGFP-empty and DsRed-empty imported into HeLa cells. The intensity analysis demonstrated that ALP (green curve) and FBLN7 (red curve) had consistent colocalization with morula and revealed a similar pattern of elevated peaks across, and adjacent to, the morula profile (Figure 4B). ALP and FBLN7 proteins exhibited both diffuse and punctate cytoplasmic colocalization in the cell.

### ALP interact with EGF domain

By Far-western blotting analysis and cotransfection assay, we identified interaction between ALP and FBLN7. The FBLN7 has been identified contain four domains (three EGF domains and one CCP domain), and the protein used in the above experiments include two EGF domain. Next, to determine if all sequences, or some special structural domain of FBLN7, were involved in specific interactions with the ALP, two other domains (EGF: 136–172 aa and CCP: 81–134 aa domains) were also tested by Far-western blotting. The results showed that specific bands were found only for the interaction of EGF domains and ALP. The CCP region of FBLN7 did not exhibit any substantial interaction with ALP (Figure 4C). This experiment confirmed that ALP interacted most specifically with the EGF domains of FBLN7.

### Recombinant ALP stimulate 3T6 cells

**Figure 5:**
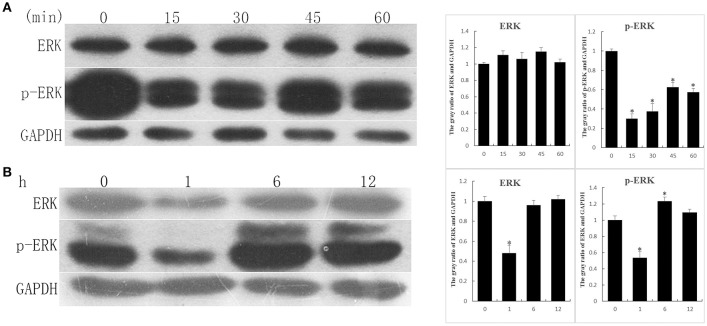
**Recombinant ALP (rALP) and *S. eriocheiris* stimulation of 3T6 cells lead to relevant protein expressions. (A)** 3T6 cells were treated with rALP (5 μg/ml) for 0, 15, 30, 45, and 60 min. The protein ERK and p-ERK variation tendencies were detected by western blotting; **(B)** 3T6 cells were treated with *S. eriocheiris* for 0, 1, 6, and 12 h. The protein ERK and p-ERK variation tendencies were detected by western blotting; GAPDH served as the control. The gray value of bands were measured. Statistical significance is indicated with an asterisk (^*^).

EGF-like domain is an evolutionary conserved protein domain, has high degree of similarity with EGF, can activate downstream pathway of EGFR signaling, in key site of amino acids. To determine whether ALP has an influence on the EGFR signaling, the 3T6 cells were treated with rALP (5 μg/ml) for 0, 15, 30, 45, and 60 min at 37°C, with 5% CO_2_. After treatment, the 3T6 cell proteins were extracted, and western blotting was used to test variation tendency of ERK and p-ERK (the main downstream pathway of EGFR signaling), using GAPDH as the control. The results showed that the ERK protein underwent no obvious changes after rALP stimulation. The p-ERK protein post rALP induction showed a significant reduction at 15 min. After that, p-ERK had a slight recovery and was also lower than the control group (Figure 5A). The variation tendency was similar to that with *S. eriocheiris*-induced 3T6 cells at an early stage (Figure 5B).

### Recombinant ALP inhibits EGF activation of EGFR pathway

**Figure 6:**
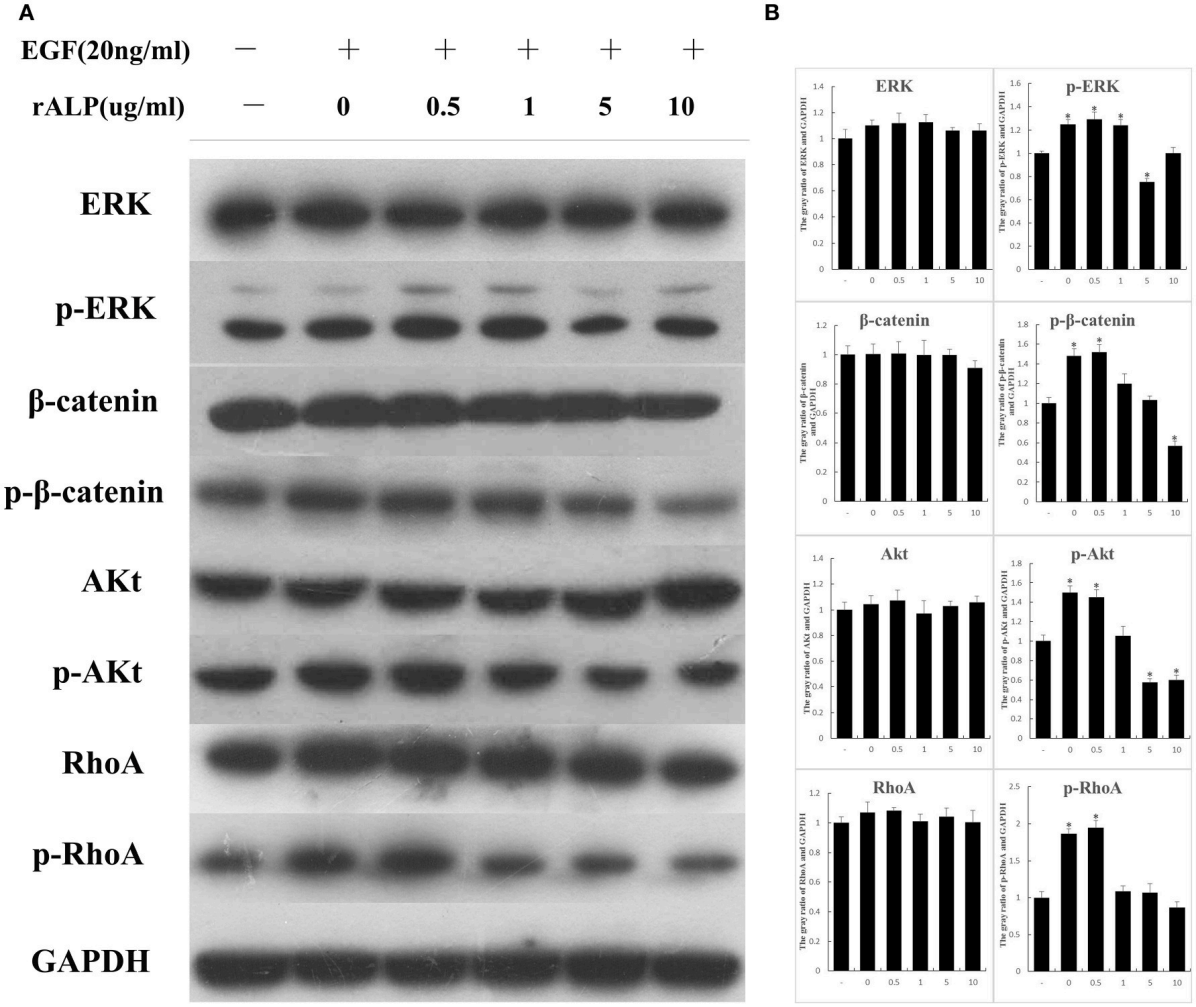
**When 3T6 cells are stimulated with EGF and treated with different concentrations of proteins, the relative protein expressions change. (A)** 3T6 cells were treated with EGF (20 ng/ml) and different concentrations of ALP protein (0.5, 1, 5, 10 μg/ml) for 15 min; untreated cells served as the control group. The protein ERK, p-ERK, β-Catenin, p-β-Catenin, Akt, p-Akt, RhoA, and p-RhoA variation tendencies were detected by western blotting; GAPDH served as the control. **(B)** The gray value of bands were measured. Statistical significance is indicated with an asterisk (^*^).

To further determined the influence of ALP on the host cells EGFR signaling. After 3T6 cells were treated with EGF and different concentrations of protein, the relative protein (ERK, p-ERK, β-Catenin, p-β-Catenin, Akt, p-Akt, RhoA, p-RhoA) expression changes were detected by western blotting. The results showed that as the rALP concentration increased, p-ERK, p-β-Catenin, p-Akt, and p-RhoA were significantly reduced compared with the control and the EGF-stimulated groups. The ERK, β-Catenin, Akt, and RhoA showed no obvious changes after stimulation, when GAPDH was used as a control (Figure 6).

### Expression of FBLN7 during mouse development

**Figure 7:**
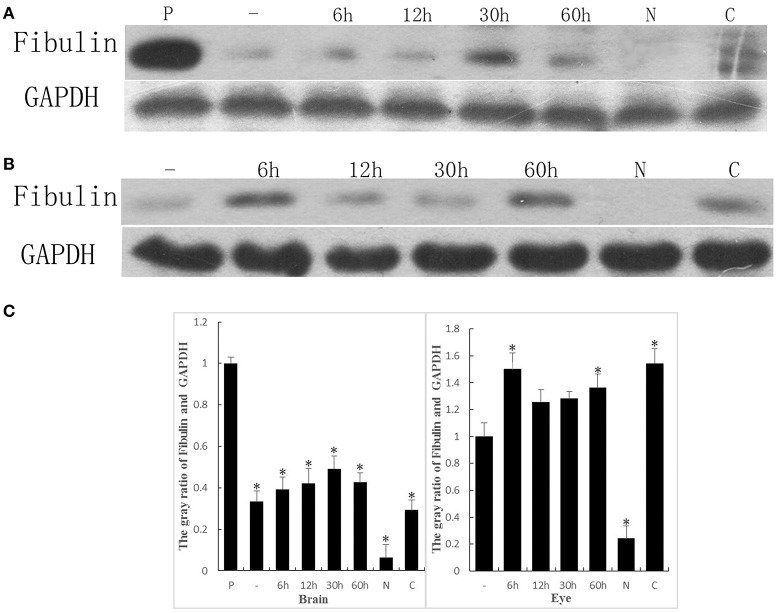
**The expression of FBLN7 during different development times of the mouse brain and eye were detected by western blotting. (A)** Brain: using placenta (P) as the positive control, unborn mouse (–), mouse postnatal from 1 to 60 h, normal adult mouse (N), and the mouse afflicted with cataracts (C); **(B)** Eye: unborn mouse (–), mouse postnatal from 1 to 60 h, normal adult mouse (N), and the mouse afflicted with cataracts (C). GAPDH served as the control, and the amount of sample used in each lane was equal. **(C)** The gray value of bands were measured. The Brain corresponding **(A)** and the Eye corresponding **(B)**. Statistical significance is indicated with an asterisk (^*^).

We have shown that ALP could interact with the EGF domain of FBLN7, thus the FBLN7 may act as one of the adhesion sites in the process of the bacteria infect host cells. In order to demonstrate that ALP interactions with FBLN7 may play an important role during the process of *S. eriocheiris* infection of the newborn mouse, we collected brain and eye samples of mice at different time points to detect the expression of FBLN7. The results showed minimal expression in the brains of unborn mice and newborn mice, while none was detected in the normal adult mouse. However, the adult mouse that was afflicted with cataract also had a small amount of expression of this protein (Figure 7A). Similar results were detected in the eye of the mouse (Figure 7B).

## Discussion

*S. eriocheiris* was previously identified as a novel pathogen of *E. sinensis* and caused mass mortality in aquaculture (Wang et al., 2004b). It is interesting that besides causing infection of crustaceans among invertebrates, *S. eriocheiris* also infected vertebrates, e.g., newborn mice, causing cataracts as the mice grew older (Wang et al., 2003); similar to an *S. mirum* infection of newborn mice (Zeigel and Clark, 1974). However, *S. mirum* had no ability to infect crustaceans (data not yet published). Evidence of *S. eriocheiris* infections in both crustaceans and newborn mice provided an interesting proposition to experimentally examine its pathogenic mechanism.

*Spiroplasma* is a Mollicute species that lacks a cell wall and does not produce endotoxins and external toxins (Stülke et al., 2009), so the outer membrane proteins of *Spiroplasma* may play an important role involved in adhesion to and invasion of host cells. Adhesion related proteins are a group of important proteins involved in the interaction between host and pathogen. The loss of adhesion capacity by mutation results in loss of infectivity, while reversion to the cytadhering phenotype is accompanied by regaining infectivity and virulence in *Mycoplasma pneumonia* (Krause et al., 1982, 1983). Loss and restoration of the ability of *Spiroplasma citri* to adhere to a monolayer of cultured *Circulifer tenellus* cells is clearly associated with degradation and restoration of a specific spiroplasma membrane protein, the spiroplasma adhesin related protein (SARP1; Yu et al., 2000; Berg et al., 2001). Furthermore, the ALP of *S. eriocheiris* shared similarities with the adhesin-related protein SARP1 of *S. citri* (Meng et al., 2010).

In this study, we demonstrated that ALP appears to be an effector protein involved in the direct interactions with eukaryotic proteins. The immune-gold labeling and western blotting techniques both confirmed that ALP is present on the outer membrane of *S. eriocheiris*. Immunofluorescence experiments show ALP was involved in the infection of host cells by the bacterium. The ALP of *S. eriocheiris* was restrained when the bacteria were incubated with anti-ALP, and therefore the ability of *S. eriocheiris* to infect host cells was limited. The ALP was confirmed to be present on the outer membrane of *S. eriocheiris* and plays a crucial role in the bacterial infection of its host cells. When the ALP was restrained, it did not completely abolish *S. eriocheiris* infection and growth; therefore, the bacterial infection of the host may be a complex process (Figure 7C). It is necessary to find a combination of the effects of several proteins or a complex to explain the invasion of host cell types by *S. eriocheiris*.

The eukaryotic target protein of ALP was identified and confirmed by the yeast two-hybrid assay, Far-western blotting, and colocalization in HeLa cells. The EGF modules of FBLN7 were the identified interacting partners with ALP. FBLN7 has been identified as a new member of the extracellular matrix proteins, with the presence of three central tandem EGF modules and a unique Sushi domain [also known as CCP module or short consensus repeat (SCR)] at the N terminus (de Vega et al., 2007). The EGF-like domain is an evolutionary conserved protein domain, containing conserved carboxylate residues and structure with the epidermal growth factor (EGF). It comprises about 30–40 amino acid residues and has been found in some of animal proteins (Bork et al., 1996; Downing et al., 1996). Most occurrences of the EGF-like domain were found in the extracellular domain of membrane-bound proteins or secreted proteins. EGF is a potent mitogenic peptide, also implicated in many non-mitogenic activities such as ion transport (Sardet et al., 1990), cellular migration (King et al., 1987), growth and development of various tissues (Carpenter and Cohen, 1997; Somboonwiwat et al., 2005), and plays a vital role in immune responses (Kansas et al., 1994; Phan et al., 2006). EGF modules are important for protein-protein interactions and bind many extracellular matrix proteins through the EGF tandem array. For instance, fibulin-1 binds to fibronectin through the EGF domain (Tran et al., 1997). At the same time, EGF-like domain also plays a key role in the process of bacterial infection of host cells. The cell wall teichoic acid (WTA) of *Staphylococcus aureus*, which directs interactions with the EGF-like domain of SREC-I, is a key factor that facilitates bacterial colonization in the nasal epithelial cells of humans (Baur et al., 2014). Similarly, the EGF-like domain was shown to interact with *S. eriocheiris* ALP in this study. It is likely that the EGF-like domain of FBLN7 binds to ALP and offer an adhesion sites for *S. eriocheiris* infects the host and accelerates this process. Because the *Spiroplasma* spp. have no cell wall, ALP may serve a role for interaction with the EGF-like domain as occurs with WTA.

In this paper, the ability of ALP to interact with the EGF-like domain was shown. And this domain containing conserved carboxylated residues and structure with EGF, was an important stimulator of the EGFR signaling. The extracellular regulated protein (ERK) kinases signal transduction pathways in 3T6 cells were restrained when cells were treated with rALP. The ERK pathway is one of the main downstream effectors of the EGFR. It may be due to a competitive activity of ALP against EGF for the EGFR site, as the EGF has a high similarity with EGF-like domain, and then as a consequence the EGFR signaling was restrained and p-ERK was decreased. When different concentrations of rALP with EGF were used to treat the 3T6 cells, the main EGFR signaling, containing RhoA/ROCK (Rho kinase), MEK (MAPK/ERK kinase)/ERK (extracellularsignal-regulated kinase), Akt (protein kinase B), and β-catenin pathways, were decreased. Previous studies have confirmed that wild-type EGFR undergoes rapid endocytosis after binding to the plasma membrane, followed by lysosomal degradation (Shtiegman et al., 2007). The small GTPase RhoA has been identified as a negative regulator of EGFR endocytosis via its effector Rho kinase (ROCK) that regulates endophilin A1-mediated crosstalk in specific cell types. Activation of the RhoA effector ROCK phosphorylates endophilin A1 at the Thr-14 amino-acid site, thus reducing the level of EGFR endocytosis (Kaneko et al., 2005). Hence, the protein competitively combines with EGF, and then the EGFR pathway is restrained upon cellular treatment with rALP; on the other hand, p-RhoA degradation results in increased EGFR internalization and therefore, the EGFR pathway is limited. When the bacteria infect the host cell, the ALP combines with EGF, thus effecting the EGFR pathway and accelerating bacterial infection of the host cell. At the same time, previous studies shown that the Akt signaling play a crucial role on the regulate of cell apoptosis. EFGR signaling play key roles in regulate cell apoptosis, cytoskeleton remodeling, cell proliferationplays and immune responses on, and so on. When *S. eriocheiris* infect the host cells the EGFR signaling were restrained, and the cells normal metabolism were destroyed, so the bacteria more likely to infect the cell (Riederer and Matus, 1985).

*Spiroplasma mirum* was confirmed to be the only species among spiroplasmas that can infect vertebrates, and it had the ability to induce cataracts in newborn suckling mice (Zeigel and Clark, 1974). Similarly, *S. eriocheiris* also can infect newborn mice and cause cataract, which is the second identified *Spiroplasma* that can infect vertebrates. Using the yeast two-hybrid assay, far-western blotting, and colocalization, we confirmed that the EGF domain of FBLN7 was the eukaryotic target protein of ALP. The prenatal, postnatal (1–60 h), and cataract mouse signals were detected at the specific band of FBLN7. There were no bands detected in any normal adult mice. These results are consistent with prior research showing that the expression levels of EGF appeared relatively stable during the early postnatal period and deeply dropped at postnatal 10 days (Lazar and Blum, 1992). This may be one of the reasons why newborn mice can be infected by *S. eriocheiris*, and the adult mouse is resistant to infection. There is abundant EGF in the mouse brain at an early stage after birth. Therefore, ALP promoted *S. eriocheiris* infection in animals by interacting with EGF. The expression levels of EGF dropped significantly several days after birth of the mice. At that point in their development, the target site of ALP was reduced. Hence, the ability of *S. eriocheiris* to infect newborn mice after several days was restricted.

**Figure 8:**
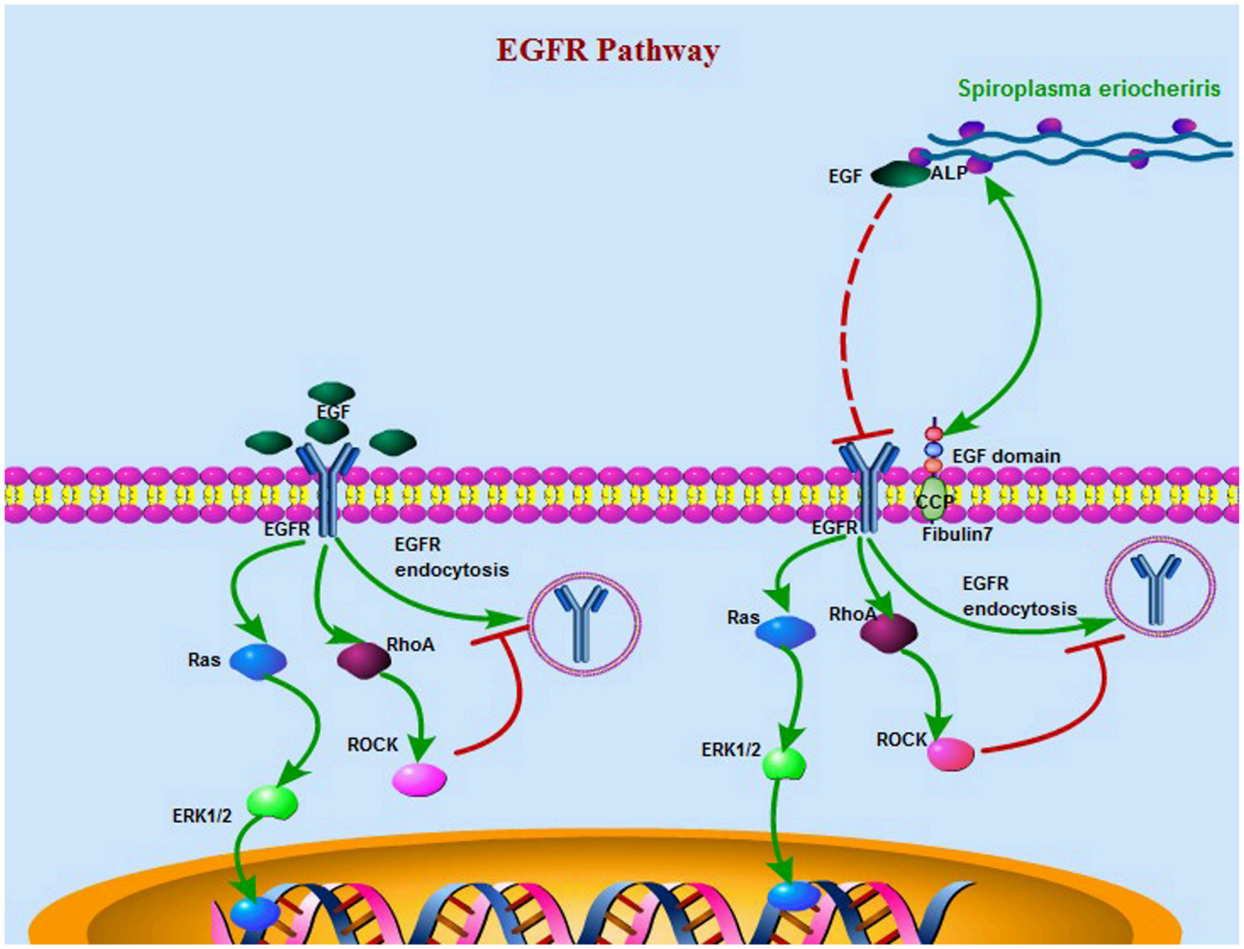
**A schematic model of the *S. eriocheiris* ALP interaction with the EGF domain protein to facilitate bacterial infection of the host cell when the EGFR pathway was effected by ALP**. On the left is the normal EGFR pathway, and on the right is the main EGFR signaling like RhoA/ROCK (Rho kinase), MEK (MAPK/ERK kinase)/ERK (extracellularsignal-regulated kinase), etc. were decreased duo to competitive binding of ALP to EGF. The small GTPase RhoA has been identified as a negative regulator of EGFR endocytosis via its effector Rho kinase (ROCK). The fibulin7 protein duo to has servels EGF domain act as an adhesion sites of the bacteria. For abbreviations and explanations, see the text.

In summary, this work advances our knowledge of *S. eriocheiris* pathogenesis by identifying ALP as a surface protein that is critical for infection of mammalian host cells. The EGF modules of FBLN7 were identified as the sites interacting with ALP. ALP interacts with the EGF domain protein to promote binding to and infection of the host cell. Simultaneously, the main EGFR signaling like RhoA/ROCK (Rho kinase), MEK (MAPK/ERK kinase)/ERK (extra cellular signal-regulated kinase), etc. were decreased duo to competitive binding of ALP to EGF (Figure 8). The unborn mouse, newborn mouse, and adult mouse with cataract had FBLN7. However, the normal adult mouse did not have FBLN7. Identification of *S. eriocheiris* effector proteins and host cell ligands such as those identified in this study will facilitate further studies to elucidate and define the molecular mechanisms involved in bacterial infections.

## Author contributions

LH, QM, WW, and WG designed experiments and analyzed the data. LH, YL, QG, MN, JB, HL, and ML performed the experiments and analyzed the data. LH wrote the paper.

### Conflict of interest statement

The authors declare that the research was conducted in the absence of any commercial or financial relationships that could be construed as a potential conflict of interest. The reviewer PT and handling Editor declared their shared affiliation and the handling Editor states that the process nevertheless met the standards of a fair and objective review

## Supplementary material

Figure S1 ***Spiroplasma eriocheiris* has the ability to infect newborn mice and cause cataracts**. The mouse on the left is a newborn pup injected with *S. eriocheiris*. The one on the right is a normal mouse.

Figure S2 **Coomasie stained PAGE gels used in the Western blots identified ALP localizes on the surface of *S. eriocheiris***. Lane M molecular-weight markers; Lane 1 membrane proteins (M), Lane 2 cytoplasmic protein (C), Lane 3 all proteins (T) of *S. eriocheiris*, respectively.

Figure S3 **Analysis of recombinant EIF and FBLN7 by SDS-PAGE**. Lane 1, soluble protein of *E. coli* BL21 (DE3) with pGEX-FBLN7 after protein expression; Lane 2, insoluble protein of *E. coli* BL21 (DE3) insoluble protein with pGEX-FBLN7 after protein expression; Lane 3, insoluble protein of *E. coli* BL21 (DE3) insoluble protein with pGEX-EIF2 after protein expression; Lane 4, soluble protein of *E. coli* BL21 (DE3) with pGEX-EIF2 after protein expression; Lane 5, soluble protein of *E. coli* BL21 (DE3); Lane 6, insoluble protein of *E. coli* BL21 (DE3); Lane M, molecular-weight markers.
