# Supplementary material for: Spiroplasma eriocheiris Adhesin-Like Protein (ALP) Interacts with Epidermal Growth Factor (EGF) Domain Proteins to Facilitate Infection
Source: Front Cell Infect Microbiol. 2017 Jan 26;7:13. doi: 10.3389/fcimb.2017.00013 (PMC5266718; doi:10.3389/fcimb.2017.00013)
Supplement: Supplementary file 1 [file DataSheet1.docx]

**The DNA sequencing data for interacting target proteins in two hybrid assays**

KLHL10 (NM025727.3)

TAACACGAGAAGATCTTTTAATACGACTCACTATAGGGCGAGCGCCGCCATGGAGTACCCATACGACGTACCAGATTACGCTCATATGAACATGGAGGCCAGTGAATTCCACCCAAGCAGTGGTATCAACGCAGAGTGGCCATTACGGCCGGGGGGAAACGAGTGCCTGTTTACAGCAGAAGTGTACAACACTGAGAGCAATCAATGGACAGTCATTGCACCCACGAGGAGCAGGAGGAGTGGAATAGGTGTAATTGCTTACGGGGAACATGTATACGCAGTGGGTGGCTTTGATGGAGCCAATCGACTGAGGAGTGCAGAAGCCTACAGCCCAGTGGCTAATACTTGGCGCACGATCCCCACTATGTTTAACCCTCGTAGCAATTTTGGGATCGAGGTAGTGGACGACCTCTTGTTTGTGGTGGGTGGCTTTAATGGCTTTACCACCACCTTTAACGTTGAGTGCTATGATGAAAGGACCGATGAGTGGTATGACGCTCATGACATGAGCATATACCGCAGTGCTCTGAGCTGCTGCGTGGTACCAGGGCTAGCCAATGTTGGGGAGTATGCAGCTAGACGGGACAACTTCACGGGATTAGCACTGCGAGATGAAGTAAAGTATTCCGCTTCGACAAGTACCCTACCTGTATGAGCCTCTTCATTTAGCTAATAAAAAGTCTAAGCAATAAGAATTAATTCTTTTTTTAAATAAATGCAGTGTTTAAACTTGAAAAAAAAAAAAAAGGCCGCCTCGGCCTCTAGAGGGTGGGCATCGATACGGGATCCATCGAGCTCGAGCTGCAGATGAATCGTAGATACTGAAAAACCCCGCAAGTTCACTTCAACTGTGCATCGTGCACCATCTCAATTTCTTTCATTTATACATCGTTTTGCCTTCTTTTATGTAACTATACTCCTCTAAGTTTCAATCTTGGCCATGTAACCTCTGATCTATAGAATTTTTTAAATGACTAGAATTAATGCCCATCTTTTTTTGGACCTAAATTCTTCATGAAAATATATTACGAGGGCTTATTCAGAAGCTTTGGACTTCTTCGCCA

PNPLA 8 (NM026164.2)

TAAAAGAAACCTTTAATACGACTCACTATAGGGCGAGCGCCGCCATGGAGTACCCATACGACGTACCAGATTACGCTCATATGAACATGGAGGCCAGTGAATTCCACCCAAGCAGTGGTATCAACGCAGAGTGGCCATTACGGCCGGGGACGTGGGAAAAGATCCTCAAGGATAGAATTGGATCTGCACTAATGATTGAAACAGCGAGAAACCCTGCATGTCCTAAGGTAGCTGCTATAAGTACCATAGTAAACAGAGGACAAACACCAAAAGCTTTTGTGTTCAGAAACTATGGTCATTTTCCTGGCACCAACTCTCACTATTTAGGAGGCTGTCAGTATAAAATGTGGCAGGCCATTAGAGCCTCATCAGCTGCTCCAGGCTACTTTGCAGAGTACGCGCTGGAGAGTGATCTTCATCAAGATGGAGGTTTGCTTCTGAATAACCCTTCGGCCTTGGCCCTTCACGAATGTAAATGCATCTGGCCAGATACCCCACTGGAGTGTATAGTATCCTTGGGCACAGGACGGTATGAGAGTGATGTGAGGAATACCTCAACATACACGAGCCTGGAGACCAAGCTCTCTAATGTTATCAGCAGTGCTACAGATACAGAGGAAGTTCATATCATGCTTGATGGCCTGTTGCCTTCTGACACGTACTTTAGATTTAATCCTGTGATATGTGAAAACATACCCCTAGATGAAAGCCGGGATGAAAAACTGGACCAGCTTCAGCTGGAAGGGATGAAGTATATAGAAAGATATGATTGGTAACACAAGTTATGTGGGAATTAGGTATTAAGATGTTCATAACCTTTAGGCATTTTATTACTGTGCTAGTGGTTTTAGTATCTATTGATCTTAAGTTGTTTACTGTTTGAATGTTTTAATATATGTGCCAAACAAGAATGGGGGATGTTACACTTCAGCCTTTGCTTTTACTCGTTTCAATCATGTGGAATTTGTGTGACTGATTTTCTTTCATGTCTTTCATTTACACTACTAGCATTTCACTAGTGCGCCACATCAATGTGTACTTTCACTGTATATCACTGAAGAGAAATAGAATGGATCTTCTTTTTAAAGTACATTTATGTTTGATATA

FBLN 7 (NM024237.4)

CAAAAAAAAAATCTTTTTAATACCGACTCACTATAGGGCGAGCGCCGCCATGGAGTACCCATACGACGTACCAGATTACGCTCATATGAACATGGAGGCCAGTGAATTCCACCCAAGCAGTGGTATCAACGCAGAGTGGCCATTACGGCCGGGGAGCTGCGAGGCGGGATTCCACCTGAGCAGCACCACGGGCGGCCACAGCGTCTGTCAGGATGTGAATGAGTGTGAGATCTATGGGCAGAAAGGACGCCCCCGGCTCTGCATGCATGCCTGTGTGAACACCCCTGGTTCCTACCGATGTACCTGCCCGAGTGGATACCGGATCCTGGCTGATGGGAAGAGCTGTGAGGATGTTGATGAGTGTGCAGGCCCACAGCACATGTGCCCCCGGGGGACCACATGCATCAACACTGGAGGAGGCTTCCAGTGTGTCAACCCTGAGTGTCCTGAGGGCAGCGGCAATATAAGCTACGTGAAGACATCTCCTTTCCAGTGCGAGCGAAACCCTTGTCCCATGGACAGCAGGCCATGCCGCCACCTGCCCAAGACCATCTCCTTCCATTACCTCTCTCTCCCTCCCAAGTTGAAGACACCCATCACGCTCTTCTGCATGGCCACAGCCTCAATTCCTGGCCATCCTGGGCCCAACAGCCTGCGCTTTGGGATCGTGGGTGGGAACAGCCGTGGCCATTTCGTAATGCAGCGCTCAGACCGGCAGACAGGAGAGCTCATCCTTACACAGACCCTGGAGGGGCCTCAGACTCTGGAGGTTGACGTAGACATGTCGGAATACCTGGAGCGCTCCTTCCAGGCCAACCATGTATCCAAGGTCACCATCTTTGTTTCTCGCTATGACTTCTGAGGATGCCATGGCAGTCGGGAGGCTGGGGTTTGAGATCTGGGCTGATTTCACTTCCCCAAGGACACATTGTGGGCAAGAGCTGTGGTGGTCATTCTTTTCTTGGTCATGTTCTTACCTACTCTGCTTGTTCATTCGGGCTTCTAGATCAATGCTGTGTCTGACCCAGGGGCTGCACAGAGGAGCCACAAACAATGCTGTTTCTACCATCAAAACAAGTAATGGCCCGCCCTCCGGT

CRA 8 (NM007592.3)

TCGGAAGGAAATTCCTTTTAATACGACTTCACTATAGGGCGAGCGCCGCCATGGAGTACCCATACGACGTACCAGATTACGCTCATATGAACATGGAGGCCAGTGAATTCCACCCAAGCAGTGGTATCAACGCAGAGTGGCCATTACGGCCGGGGGGAAAATCCAAAACAATTCCATGCTTTAATCCTAACACTTTATTACCAGACCCTCTTCTGCGGGATTACTGGGTCTATGAAGGATCTCTTACTATCCCACCTTGCAGCGAAGGAGTTACCTGGATATTATTCCGATACCCTTTAACTATATCCCAGATGCAGATAGAAGAATTTCGAAGGCTGAGGACACATGTCAAGGGGGCAGAACTGGTGGAGGGCTGCGATGGGATTTTGGGAGACAATTTCCGACCTACCCAGCCCCTGAGTGATAGAGTCATCCGAGCGGCCTTTCAGTAGCCAGAGAGACCGGGAACAAACCCATCTGCATCAGTGAAGAAGACAGTGGTCCCACGGAGTTTTTTGATGAGGAGTGGAAACTCCTGAGTGGCAGCTTTGTTTTAACCCTCAAGCCTGCACTATCTTCAGTTACTGCAGTGTTGATGGATATCTGTGACCATTGTCTGTATACATGCTGTTATGAAATATTAGAAGTGGCTGTTCATTGTAAAGAAACACAGTGTGTGTGCACACACCACAGCTTCTGGAATTCATACTTTTGCACATACTGCTTGTTGCTTATGTGTAGAAAGAATGAAAGTAGTTTTTCAGACATACTAATATGACTGTATTATATATCAATATGAGATAGGAAAAGCACATTCCCAGAGCCAAGTCATTTATTTCCTGTCTGTATTAGAAAATATGCTCACTAGTGTATTTCTTGATAATAAAGCAAGATGTTTGATCTACAACAGCTTAGAACATCAAAATACATATGAAGGAGTCATAAAAATTATCTCGTGTTGTTTTGCATAATTGCACCAATTTAATGTCAACTGGTTTGTCATGAAAAATTTGAGTATGCAAGATACTTTATATATATTTTTTAGAGATTTATGATCTAATATACTTATGGCATATTCTTGATGTATGGCTTTAGGAGTA

GSg 1 (NM010352.2)

AAAGTGCGCTTATTTTAATACTACTCACTATAGGGCGAGCGCCGCCATGGAGTACCCATACGACGTACCAGATTACGCTCATATGAACATGGAGGCCAGTGAATTCCACCCAAGCAGTGGTATCAACGCAGAGTGGCCATTACGGCCGGGGGGCATTCATCCTCTCCCAACTGCCCACATACCACCTTACCCCCTCTGCTGGCAAGAGGGGACCTGGTGCACCCTCTCCCTCATGTCACCCCACTCTCCGATTTGGAGGGGAGTGGTTAATGGAGAAGGCTTCTCTCCTCCACCTCCCTTGGGGGCCCGTGGCAAAGGTCTTTTGGCTGTCACTGGGAGCCCAGACTGCCTATATCGGACTTCAACTCATCAGCTTCCTCCTGCTACTGACGGATCTGTTGCTCACCAAGAACCCTGGCTGTGGGCTCAAGCTAAGCGCGTTTGCAGCCGTCTCCTTGGTCCTGTCAGGACTTCTGGGGATGGTGGCTCATATGCTATATTCACAAGTCTTCCAGGCAACTGCCAACTTAGGTCCGGAGGACTGGAGACCACACTCTTGGAATTACGGCTGGGCCTTCTACACAGCGTGGGTTTCCTTCACCTGCTGCATGGCGTCAGCGGTCACCACCTTCAACATGTACACGAGGATGGTGCTGGAGTTCAAGTGCAGGCACAGCAAGAGCTTTAACACCAACCCCAGCTGCCTGGCGCAGCACCACCGCTGTTTCCTTCCTCCTCCGCTGACGTGCGCAACCCACGCAGGGGAACCTTTGTCCAGCTGCCATCAGTACCCCAGCCATCCCATCCGCTCTGTCTCTGAAGCTATTGACCTCTACTCGGCGCTACAGGACAAAGAATTTCAACAAGGGATCAGCCAGGAGCTAAAGGAAGTGGTCGAGCCATCTGTAGAAGAGCAGCGTTAGGAGTTAAGTGGGTTTGGGAAGCAGCTAAGTCCTACCATAGTGTCGCTCACTTTCAACATCTGCTTAAGCAAAAAAAAAAAAAAGGCCGCCCTCGGCCTCTAAAGGGTGGGCATC

LMPA 2 (NM053261.2)

TACAAAATATAAGTTTTCTACGACTCACTATCTCGGCCAGCGCCGCCATGGAGTACCCATACGACGTACCAGATTACGCTCATATGAACATGGAGGCCAGTGAATTCCACCCAAGCAGTGGTATCAACGCAGAGTGGCCATTACGGCCGGGGGAAGATTTAATTGTTTCTGAGTTGCGAAAGCGGTTTCCTTCACACAGGTTCATTGCAGAAGAGGCCACAGCCTCCGGGGCCAAGTGTGTGCTCACCCACAGCCCGACCTGGATCATCGACCCCATCGACGGCACCTGCAACTTTGTGCACAGGTTCCCCACTGTGGCAGTTAGCATCGGATTTGCTGTTCACCAGGAGCTGGAATTTGGAGTGATTCACCACTGCACAGAGGAGCAGCTCCACCTCGGCCCTCTGTTACCTGGCCTCAGGTGCAGCCGACGCCTATTATCAGTTTGGCCTTCACTGCTGGGACCTGGCAGCTGCCACCGTCATCATTAGAGAAGCAGGCGGCATTGTGATTGACACCTCAGGTGGACCCCTTGACCTCATGTCGTGCAGAGTCGTAGCTGCTGGCACCAGAGAGATGGCAGTGCTCATAGCTCAGGCCCTACAAACTATTAACTACGGCCGAGACGATGAGAAGTGAGCCACACACAGCTCGAAGGCTAAAAACGCAGCAGCAACCTGGGAAAGAGCTGTCCCGGTGGCTTAAGTTCCAGGACAGTCTACCATAGCTCTCCCTGGGCCTTGCCTCGGTGCTTAGCTGATTCTCTCTAATCTCGTGTAGCCCCTTTTCAGGTCGGTACGTGTTCTTTTCATCAGAGCCAAACCCAGATCTTGTGAGGGGTGTGTTAGTCATCCATCCTGATTGTTTTTCCAGAATGCAAATCTCATGTGATACAGCTTTAGAACGGGCTCTCAGGCTCTCCCCTGACCGTGGTAAGACGGAATGCAATAAATCAGAATTATAGTGGTCAA

COPS 5 (NM013715.2)

AAAAAAGAACTTTTTAATACGACTCACTATAGGGCGAGCGCCGCCATGGAGTACCCATACGACGTACCAGATTACGCTCATATGAACATGGAGGCCAGTGAATTCCACCCAAGCAGTGGTATCAACGCAGAGTGGCCATTACGGCCGGGGATCGATGAAACCTACAAATATGACAAAAAACAACAACAAGAAATCCTGGCGGCGAAACCCTGGACTAAGGATCACCACTACTTTAAATACTGCAAAATCTCAGCATTGGCTCTACTGAAAATGGTGATGCATGCCAGGTCAGGAGGCAACTTGGAAGTGATGGGTTTGATGCTCGGGAAAGTCGACGGCGAGACCATGATCATCATGGACAGTTTCGCTTTGCCTGTAGAGGGCACAGAAACTCGAGTAAATGCTCAAGCTGCTGCGTATGAGTATATGGCTGCATACATAGAAAATGCCAAACAGGTTGGCCGCCTTGAGAATGCAATCGGTTGGTATCATAGCCACCCTGGTTATGGCTGCTGGCTCTCCGGGATTGATGTTAGTACACAGATGCTGAACCAGCAGTTTCAAGAACCATTTGTAGCAGTGGTGATTGATCCAACCAGAACAATCTCTGCAGGAAAAGTGAATCTTGGCGCCTTTAGGACATATCCAAAGGGCTACAAACCTCCTGATGAGGGACCTTCTGAGTACCAGACTATCCCACTTAATAAAATAGAAGATTTTGGCGTGCACTGCAAACAATATTATGCCTTAGAAGTCTCATATTTCAAATCATCTTTGGATCGTAAACTACTTGAGCTTTTGTGGAATAAATACTGGGTGAATACCCTGAGCTCCTCTAGCTTGCTTACTAATGCAGACTACACCACAGGCCAGGTGTTTGATTTGTCTGAGAAGTTAGAGCAGTCGGAAGCCCAACTGGGACGTGGCAGTTTCATGTTGGGCTTAGAAACACATGACCGCAAGTCGGAAGACAAACTTGCCAAAGCTACTAGAGACAGCTGTAAACCACCATAGAAGCCATCCATGGACTGATGTCTCAGTTATAGATAAACTGTTATCAGATTACGTGCTTAGTTACCACCCAGTACTTCTCAAGCTGGTGTTGTGGAAGGAAAAGAAGCCTCAGGTAAACACTT

XPNPEP 1 (NM133216.3)

TCAAGGAGAAAGTCTTCTTTCTGAATCTCTTTAGCGGCGAGCTCCGCCATGGAGTACCCATACGACGTACCAGATTACGCTCATATGAACATGGAGGCCAGTGAATTCCACCCAAGCAGTGGTATCAACGCAGAGTGGCCATTACGGCCGGGGGGACATGGCGTTGGGTCCTTTTTGAATGTTCACGAGGGGCCCTGTGGCATCAGTTATAAAACATTCTCCGATGAGCCCTTGGAAGCGGGCATGATCGTCACTGATGAGCCAGGGTATTACGAAGACGGGGCATTTGGGATCCGCATTGAGAATGTTGTTCTGGTGGTCCCAGCAAAAACCAAGTATAATTTCAACAACCGAGGAAGCCTGACCTTTGAACCTCTAACTTTGGTTCCCATCCAGACCAAAATGATAGATGTGAATGCTCTTACAGATAAAGAGTGTGACTGGCTCAACAGCTACCACCAGACCTGCAGGGACGTGGTTGGGAAGGAGCTGCAGAGCCAGGGCCGCCAGGAAGCTCTCGAGTGGCTCATCAGAGAGACAGAGCCTGTCTCCAGGCAGCATTGATGTCGCCTGGCGTTGGTTTTTCGTAGGATGCTCTGGGGGAAGGAGGACACAAGGCAGACCCCTGACTTCTCTCTCCTCACCTCCTCCTCTTCCCCGACTCCTCTTTTTACTTTTAGACACTAAGAAGAGCTGAAACTCTTCTTACCTACTTTGATATTTTCTTGCAAACAGTCTTTTATGAATTTTTAATTGTTGAGAATGAGCCAGGAATAAAACCGCTACACCAGAAGGAGGGGCCCCACGAAGCCGAAGACTTGACAAGGGGGAGACACCCCAAGCCCTCTGGCCAGGGATGGCCAACACTGACTGCTCCGCGATGGTCTCGTTCCAGGTGCTAGGACCTCAGTCACGGTCACCTTGATGTTCATGAGACCCTCCTATGATCAGTGAATAAAACCATCAAAACTCAAAAAAAAAGAAAAAAAAAAAAAAAAAGGCCGCCTCGGCCTCTAGAGGTGGGCATCGATACGGGATCCATCGAGCTCGGAGCTGCAGATGAATCGTAGATACTGAAAAACCCCGCCA

MCCC 2 (NM030026.2)

AAATAACCAAACTTCACTATAGGGCGAGCGCCGCCATGGAGTACCCATACGACGTACCAGATTACGCTCATATGAACATGGAGGCCAGTGAATTCCACCCAAGCAGTGGTATCAACGCAGAGTGGCCATTACGGCCGGGGAGAGACTATGAAGCTGAAGGGATTGCCAAGGATGGTGCCAAAATGGTAGCTGCAGTAGCCTGTGCCAAAGTGCCTAAGATAACTGTCATAATTGGGGGATCGTACGGGGCTGGAAACTACGGAATGTGTGGCAGAGCATACAGTCCAAGGTTTCTCTACATGTGGCCAAATGCCCGCATCTCTGTGATGGGAGGAGAGCAGGCAGCCACGGTGTTAGCCACAGTGGCTAGAGACCAGAAAGCACGCGAGGGGAAGCAGTTCTCCAGTGCAGAGGAGGCAGCTTTGAAAGAACCCATCATTAAGAGGTTCGAAGAGGAAGGGAACCCTTACTACTCTAGTGCAAGGCTGTGGGACGATGGGATTATTGACCCGGTGGACACCAGGCTGGTCCTGGGGCTCAGCCTCAGTGCAGCCCTCAATGCGCCCATCCAGAGGACTGACTTTGGCATCTTCAGGATGTAACTCAGATATGGAACATGCTGCCACACCCGTAACAAGAATTAGCGCGTGTAACTAGCCTTGAAACTTGAGATTTACTGGACATGTAGCTGTCACAGGAATTCTTTTCTTAACAGTATGATTTGTTAAAACTGTGAAAATCAGTGACTAGTCTGCCTGATGAGCTTTGACTTTTTTTTTTAAAAAAAAATTTTTTTCTTACAGACATTTTTCACGGCTCATTTTTACCACCCATAAAATGAAGAGAGCGATTTTGCAATTATCCTTTGAGTACCACAAACAGTATGAAAAATAAATCTCTTTAATCTGTAC

EIF 2 (NM023502.1)

CACACATATCTTTTAATACGACTCACTATAGGGCGAGCGCCGCCATGGAGTACCCATACGACGTACCAGATTACGCTCATATGAACATGGAGGCCAGTGAATTCCACCCAAGCAGTGGTATCAACGCAGAGTGGCCATTACGGCCGGGGAGGACAGTTCGTGTGGCAATGCAAGTCCCTGTGGTAATGACCTCACTGGGCCAAAAGATTTCAGCGGTGGCAGTTCAGTCAGTCAATGCAGGCACAGGCTCGCCGTTAATAACCAGCACCAGTCCAGCCTCGGCCAGCTCTCCAAAAGTAGTCATCCAGACAGTCCCGACCGTGATGCCGGCCTCCACTGAAAATGGAGACAGAATCACCATGCAGCCTGCCAAGAGTATCACCATCCCCGCCACCCAGCTCGCACAGTGTCAACTCCAGGCAAAGTCAAACCTGACGGGGTCAGGAAGCATTAACATTGTTGGAACCCCACTGGCTGTGAGGGCACTCACCCCTGTTTCAATAGCCCACGGTACACCTGTAATGAGACTGTCTGTGCCTGCTCAGCAGGCTTCTGGCCAGACTCCTCCTCGAGTTATCAGTGCGCTCCTAAAAGGGCCGGAGGGGAAATCAGAAGCCGAAAAGCAGGAACACGATGTGAAAACTTTGCAGCTGGTAGAAGAGAAGGGGGCAGACGGCAATAAGACAGTAACCCACGTAGTGGTCGTCAGTGCGCCGTCTGCTATCGCCCTTCCTGTGACTATGAAAACCGAAGGGCTAGTGACGTGTGAGAAATAAATAAGCACGTTCCGGGCGGGCACTGCAGACTGTCAGGGTTGTCCTGACAGACATTTGCAAGGGAGTCATCAAGAAAGCCAGAGGAGGGTTCTCACACAACTGTGCATAGAAGAAAGCAATCAGACGTACTGGAAGTAAATGACCTATCCCATGTTTCAGTGGGAAGTGAACTACACATTGAGATGCTGACAGAAAACTGCCTCTTAAGTAAGAACAACTGAACCCTCAATAAGAAAAGCCTGAAG

YrDC (NM153566.2)

ACCTAACCTTTCACTTATTAAGGGCGAGCGCCGCCATGGAGTACCCATACGACGTACCAGATTACGCTCATATGAACATGGAGGCCAGTGAATTCCACCCAAGCAGTGGTATCAACGCAGAGTGGCCATTACGGCCGGGGCTGGGCCGCGTGGCCGACGTCTACAGGTACTGTCAGGTGAGAGTACCTAGGGAGCTCCTGGAAGACCTGTTCCCAGGCCCTGTGACCCTGGTGATGGAGCGCTCCGAGGAGCTCAACAAAGACCTGAACCCCTTTACTCGTCTTGTTGGCATCCGGATTCCTGACCATGCCTTCATGCTGGACTTGGCCCAGATGTTTGGGGGACCACTTGCACTCACTAGTGCCAACCTCAGCTCCCAGGCCAGTTCTCTGAGTGTTGAGGAGTTCCAAGACCTCTGGCCTCATTTGTCCCTTGTCATTGGTGGGGGGCCAATTGGGGATAGTCAGAGCCCTGAGTGTCGCCTCGGCTCTACTGTGGTTGACTTATCTGTGCCTGGAAAGTTTGGCATTATTCGCCCAGGCTGTGCCCTGGAAAACACTACATCGATCCTCCAGCAGAAATATGGGCTGCTCCCTTCACAGGGGTCCTGTTCATGAAACTTGGGAGGACCCAAGGACCATGCTGGATACTATGTGTCTGCTACTGGATGGCAAAGCCTCATTGCCTGAGGTTCCTACATCTATAGCCTAGCTTTTTAGGCAGCATCCTTGGCTCTGAATCCTGTAGGCCAGCCAGAAGCTGCAGGGTGAGCTTTGTCCCCGGGGGAAGGTTATATTTATTTATTCTGTACATTTATTTGTCAGCCAAGAATTAAATGGAGGGAAGGATGTTCTTAGAGTGGCCTTATTATTTTAAGTGCCCCCTCTCACCCCCACCCCCTTTAAGTAACTTGAGTACAGAATTAGAATGCATTAAAAGCTGCTTTCTGGGGAACAGTGACATTTGATGTCAAACCAGCCAGAAGCACTAATGCAGTCTAGAATAGAAGTCTTAGGACCAACGCAGCAAAGTCTAGGAGCCGAA

FOXred 1 (NM172291.1)

TAAAAGAAATCTTTTTATACGACTCACTATAGGGCGAGCGCCGCCATGGAGTACCCATACGACGTACCAGATTACGCTCATATGAACATGGAGGCCAGTGAATTCCACCCAAGCAGTGGTATCAACGCAGAGTGGCCATTACGGCCGGGGAATGTCCATGTGAAAATGGACAAGAGCCTGGAGTATCAACCAGTAGAATGTGCTGTAGTGATCAATGCTGCAGGAGCCTGGTCTGGGAAAATTGCAGAGCTGGCTGGTGTTGGGAAGGGACTTCCTGGCACCCTCCAGGGCACCAAGCTACCTGTGGAGCCAAGGAAAAGGTATGTGCACTTATGGCACTGCCCACAGGGACCAGGTCTGGAGACACCGTTGGTTGCAGACATTAGTGGAGTCTATTTCCGACGGGAAGGATTGGGCAGCAACTACCTAGGTGGCTGTAGTCCTACTGAGGAGGAAGAACCAGACCCAACAAATCTGAATGTGGACCATGACTTCTTCCAGAACAAAGTGTGGCCTCATTTGGTCCAGAGGGTGCCATCTTTTAAGACTCTGGAGGTACAGAGTGCCTGGGCTGGTTATTATGACTACAATACTTTTGACCAGAATGGCGTGGTGGGCCCCCACCCACTAGTTGTCAACATGTACTTTGCTACGGGCTTCAGTGGTCGTGGGCTTCAGCATGCACCTGGCATCGGACGCGCTGTGGCAGAGATCATGCTAGAGGGTCACTTTAAGACCATCGACATGAGCCCCTTCCTCTTTACCCGCTTTTACTTAGGAGAGAAGTTACAGGAGTACAATATCCTCTGAGTGTGAAAACTGTCTCTGGGCCTCACCAGCCCAGGCATCACTCTGTCTCACGCTGGCTTTGGTCACTGTCTGAGTCTCCCCAGCACTGTGCCAGGGATACATCCCTTTCTTCACCATCCCTTAAACCAGGCCTCTTCCCCGGTGTCTCTGGACATGGAATGAGCATAGGCACAAAGACCCAGACCAGTGGAAGTGATGAGTGGGAGCCTAGGACTGATTGTAGCCTAGCTGCTTCACTACCTAAACAGTTTCAGCTGGCCATCCAGCATGATGCCCAGACTGCCTCCTTTCCTGGTGCTGATGCAGAAGAAATCTGACC
