# Supplementary figures and images for: Spiroplasma eriocheiris Adhesin-Like Protein (ALP) Interacts with Epidermal Growth Factor (EGF) Domain Proteins to Facilitate Infection
Source: Front Cell Infect Microbiol. 2017 Jan 26;7:13. doi: 10.3389/fcimb.2017.00013 (PMC5266718; doi:10.3389/fcimb.2017.00013)

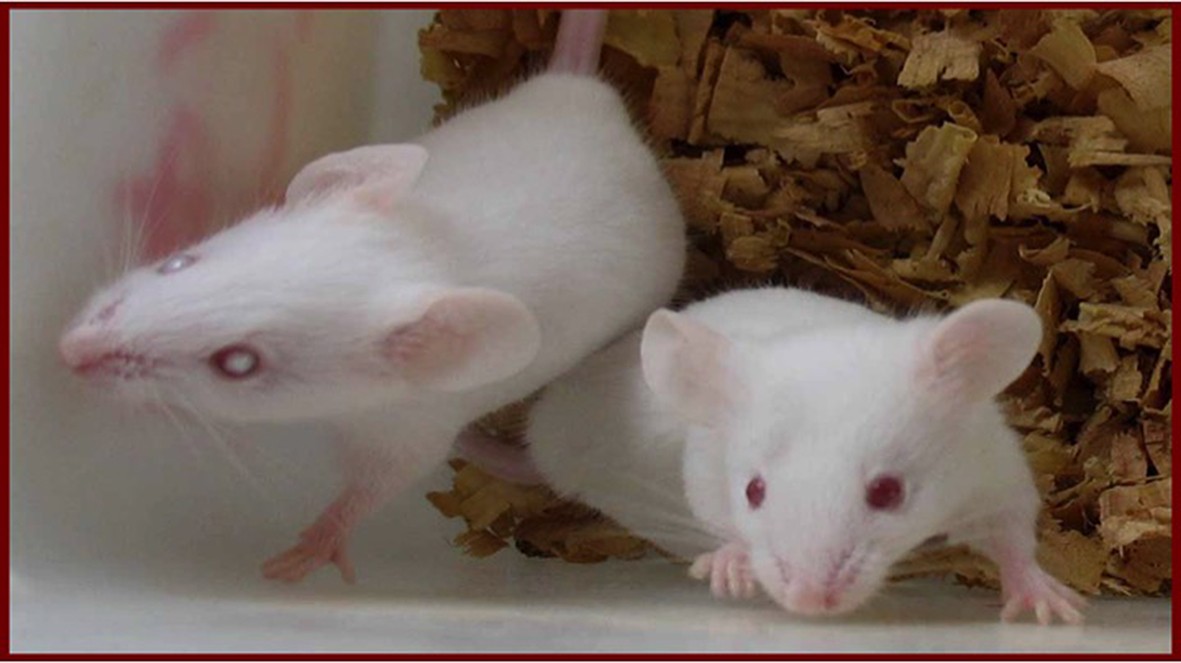

Supplement: Figure S1 — Spiroplasma eriocheiris has the ability to infect newborn mice and cause cataracts. The mouse on the left is a newborn pup injected with S. eriocheiris. The one on the right is a normal mouse. [file Image1.JPEG]

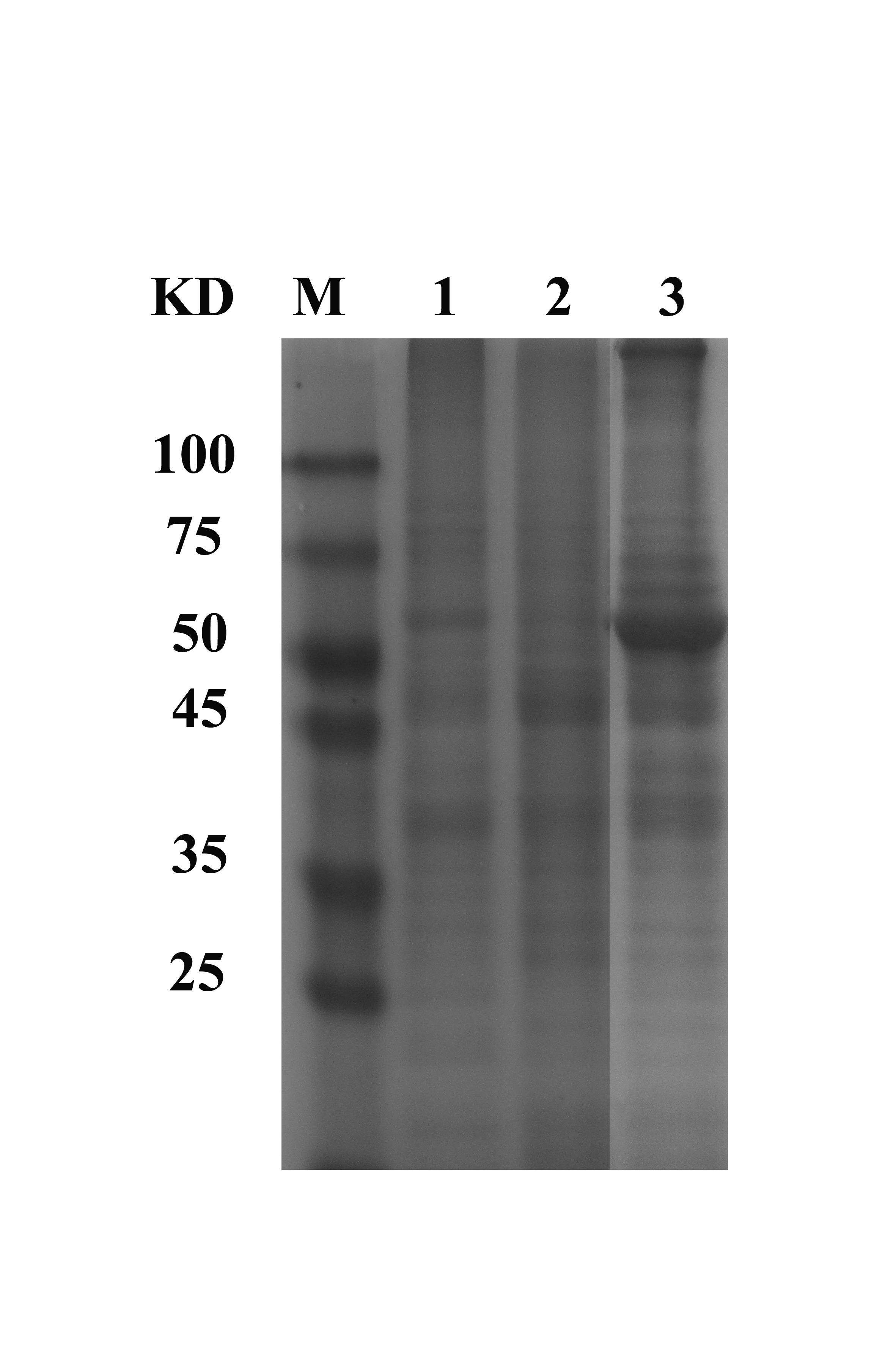

Supplement: Figure S2 — Coomasie stained PAGE gels used in the Western blots identified ALP localizes on the surface of S. eriocheiris. Lane M molecular-weight markers; Lane 1 membrane proteins (M), Lane 2 cytoplasmic protein (C), Lane 3 all proteins (T) of S. eriocheiris, respectively. [file Image2.JPEG]

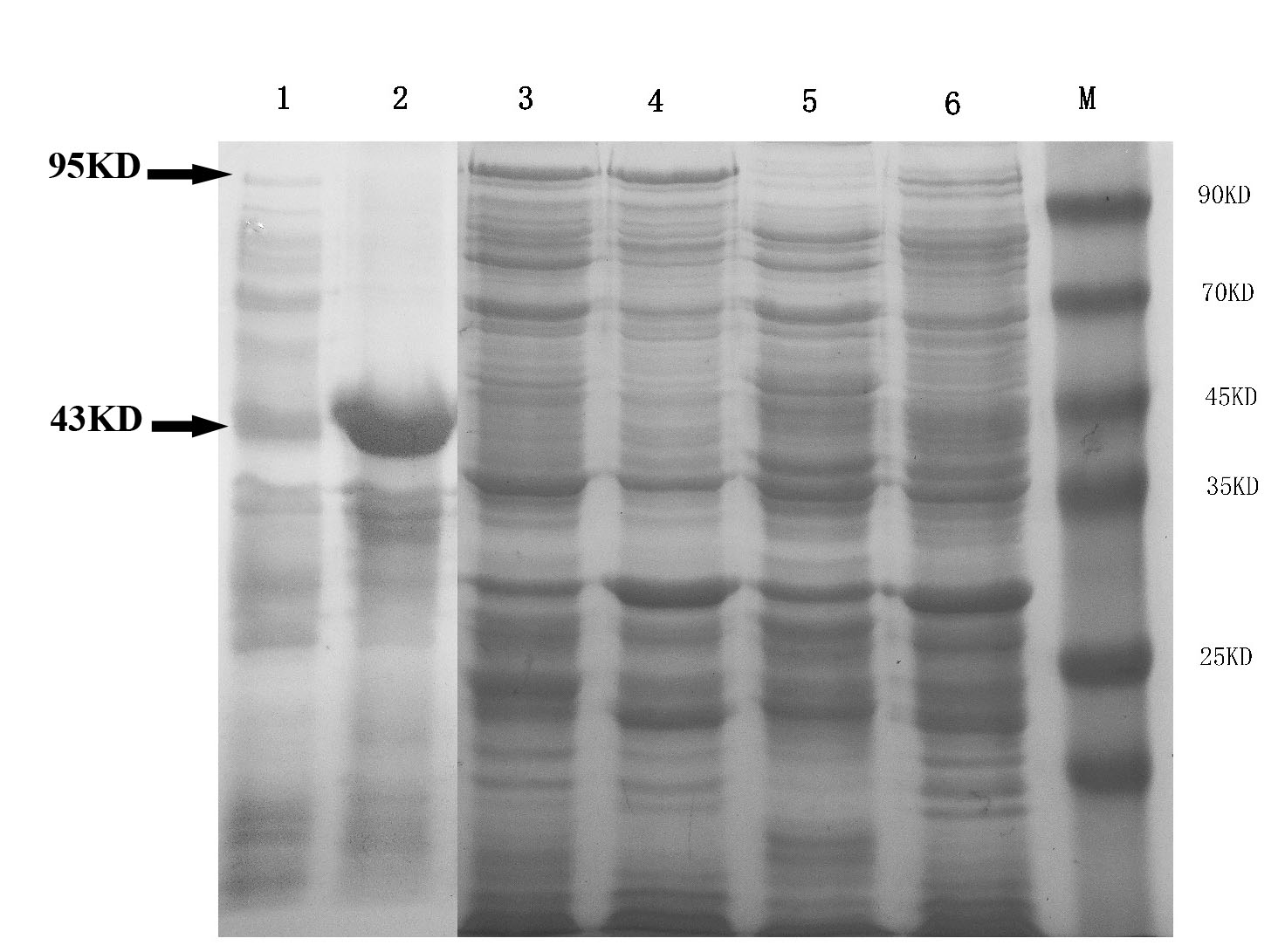

Supplement: Figure S3 — Analysis of recombinant EIF and FBLN7 by SDS-PAGE. Lane 1, soluble protein of E. coli BL21 (DE3) with pGEX-FBLN7 after protein expression; Lane 2, insoluble protein of E. coli BL21 (DE3) insoluble protein with pGEX-FBLN7 after protein expression; Lane 3, insoluble protein of E. coli BL21 (DE3) insoluble protein with pGEX-EIF2 after protein expression; Lane 4, soluble protein of E. coli BL21 (DE3) with pGEX-EIF2 after protein expression; Lane 5, soluble protein of E. coli BL21 (DE3); Lane 6, insoluble protein of E. coli BL21 (DE3); Lane M, molecular-weight markers. [file Image3.JPEG]
